# Disrupted thalamocortical functional connectivity and canonical resting-state network integration in posttraumatic stress disorder

**DOI:** 10.1016/j.nicl.2025.103927

**Published:** 2025-12-12

**Authors:** Nick Steele, Ahmed Hussain, Delin Sun, Courtney Russell, Ashley A. Huggins, Nicholas D. Davenport, Seth G. Disner, Scott R. Sponheim, Thomas Straube, David Hofmann, Shmuel Lissek, Hannah Berg, Daniel W. Grupe, Jack B. Nitschke, Richard J. Davidson, Ruth Lanius, Maria Densmore, Jean Théberge, Richard W.J. Neufeld, Sophia I. Thomopoulos, Paul M. Thompson, Rajendra A. Morey

**Affiliations:** a Brain Imaging and Analysis Center, Duke University, Durham, NC, USA,; b Department of Veteran Affairs Mid-Atlantic Mental Illness Research, Education and Clinical Center, Durham, NC, USA; c Department of Psychiatry and Behavioral Sciences, School of Medicine, Duke University, Durham, NC, USA; d Department of Psychology, University of Arizona, Tucson, AZ, USA; e Minneapolis VA Health Care System, Minneapolis, MN, USA; f Department of Psychiatry , University of Minnesota, Minneapolis, MN, USA; g Institute of Medical Psychology and Systems Neuroscience, University of Münster, Münster, Germany; h Department of Psychology, University of Minnesota, Minneapolis, MN, USA; i Center for Healthy Minds, University of Wisconsin-Madison, Madison, WI, USA; j Department of Psychiatry, University of Wisconsin-Madison, Madison, WI, USA; k Department of Psychology, University of Wisconsin-Madison, Madison, WI, USA; l Department of Neuroscience, Western University, London, ON, Canada; m Department of Psychiatry, Western University, London, ON, Canada; n Imaging Genetics Center, Mark and Mary Stevens Neuroimaging & Informatics Institute, Keck School of Medicine of USC, Marina del Rey, CA, USA

**Keywords:** Thalamic nuclei, Pulvinar, Mediodorsal thalamus, PTSD, Large-scale networks, Resting-state functional connectivity

## Abstract

The thalamus exhibits widespread connectivity to the entire cortical mantle, yet distinct thalamic subregions possess unique connectivity profiles and functional roles. While the thalamus has been consistently implicated in posttraumatic stress disorder (PTSD), fine-grained investigations examining thalamic subregions and nuclei remain sparse. We examined how resting-state functional connectivity (RSFC) of thalamic nuclei with the cortex and large-scale brain networks may contribute to PTSD using high-resolution functional magnetic resonance imaging (fMRI) data from a multi-site dataset of PTSD cases and controls (*n* = 397). We show that the pulvinar nuclei exhibit weaker RSFC with sensorimotor and salience regions, while the medial geniculate nucleus (MGN) exhibits stronger RSFC with the sensorimotor cortex in PTSD. Greater PTSD severity correlated with weaker RSFC between both the pulvinar and mediodorsal thalamus and cortical sensory/motor regions in the frontal, parietal, and occipital lobes. We identified that the default mode network of PTSD participants had stronger RSFC with the mediodorsal thalamus, while the salience and somatosensory networks exhibited stronger RSFC with somatomotor thalamic nuclei. Fine-grained thalamic mapping is important for uncovering thalamocortical disruptions in PTSD. Thalamic RSFC shows a shift toward heightened subcortical sensory responsivity and diminished voluntary control and cognitive regulation in PTSD.

## Highlights

• Examined thalamic nuclei RSFC in a large multi-site PTSD dataset (n = 397)
• PTSD linked to weaker pulvinar/mediodorsal RSFC with sensorimotor/salience regions.
• Mediodorsal thalamus displayed stronger RSFC with the default mode network in PTSD.
• Somatosensory thalamus displayed stronger RSFC with somatosensory network in PTSD.
• Results suggest a shift toward heightened sensory responsivity in PTSD.

## 1 Introduction

Posttraumatic stress disorder (PTSD) is a complex psychiatric condition that has been increasingly linked to thalamic disruption (Jeon et al., 2020, Kim et al., 2007, Morey et al., 2008, Morey et al., 2015, Yin et al., 2011, Zhang et al., 2016). After exposure to a trauma, individuals who develop PTSD often experience profound changes in emotion regulation, mood, sensory reactivity, and a variety of somatic and cognitive dysfunctions (American Psychiatric Association, 2013). Thalamocortical loops and thalamic connections with subcortical and brainstem structures mediate crucial sensorimotor, limbic, and cognitive brain functions, which may relate to psychiatric symptoms, including those observed in PTSD.

The thalamus is a central processing hub in the human brain, integrating information from diverse cortical and subcortical regions and sending widespread efferent projections to the entire cortical mantle (K. Hwang et al., 2017, Kawabata et al., 2021). Neuroimaging research involving the thalamus has often treated it as a homogeneous structure. However, the whole thalamus may not serve as the most informative unit of analysis for understanding psychiatric conditions as it comprises a diverse array of subregions, each with distinct functional roles and connectivity profiles. For example, the medial and lateral geniculate transmit auditory and visual input, respectively, and are involved in rapid threat detection (Fratzl et al., 2021, Gu et al., 2025); while nuclei within the ventral subregion function to integrate and relay motor commands and somatosensory information (Sommer, 2003, Song and Semework, 2015). Cognitive and affective processing are predominantly carried out by higher-order nuclei. The pulvinar nuclei support visual attention, multimodal sensory integration, and salience processing (Barron et al., 2015, Cortes et al., 2024) and have been implicated in PTSD pathophysiology via weaker connectivity with parietal and sensorimotor regions (Terpou et al., 2018). The mediodorsal nucleus is involved in memory-related and affective processing, including the acquisition and extinction of fear memories, via its dense connections with the prefrontal cortex (Lee and Shin, 2016, Li et al., 2022). Prior work has found altered structural properties, including smaller volume and distrupted structural covariance, of the mediodorsal thalamus in PTSD (Steele et al., 2025a, Steele et al., 2025c). This functional heterogeneity suggests that distinct thalamic circuits may differentially contribute to core PTSD symptoms such as hyperarousal, emotional dysregulation, and altered sensory processing.

Mounting evidence points to the thalamus as a key node in circuits that contribute to a range of psychiatric illnesses (W. J. Hwang et al., 2022), including stress- and trauma-based psychopathologies like PTSD. Neuroimaging studies of individuals with PTSD have provided evidence of dysfunctional activation and connectivity of the thalamus, including reduced cerebral blood flow (Kim et al., 2007) and altered connectivity with medial frontal, anterior cingulate, and sensorimotor cortices (Jeon et al., 2020, Yin et al., 2011, Zhang et al., 2016). However, investigations of the whole thalamus have obscured potential differences across the functionally heterogeneous set of thalamic divisions. To elucidate the role of the thalamus in psychiatric disorders, fine-grained investigations targeting specific thalamic subregions and nuclei are needed.

Furthermore, the extensive and diverse connectivity of the thalamus with the cortex facilitates complex transthalamic cortico-cortical communication (Guillery, 1995, Sherman, 2016), enabling the integration of sensory, cognitive, and affective information within and between distributed networks. This architecture also enables the thalamus to influence the spatiotemporal evolution of network activity (Cabrera-Álvarez et al., 2023; K. Hwang et al., 2017, Shine, 2021). These functional roles suggest that, beyond regional disruptions in thalamocortical connections, thalamic nuclei may exert varying effects on large-scale resting-state networks (RSNs) in PTSD. Altered connectivity patterns in PTSD have been observed in key networks such as the default mode and salience networks (Koch et al., 2016). Demonstrating that well-localized thalamic subregions exhibit altered functional coupling with large-scale brain networks may provide insights into how network dynamics are impacted by PTSD.

Leveraging high-resolution 3 T functional magnetic resonance imaging (fMRI) data from six study sites contributing to ENIGMA PTSD (*n* = 397), we examined thalamic nuclei RSFC differences in PTSD. We hypothesized that PTSD participants would exhibit weaker RSFC between the pulvinar nuclei and both sensorimotor and parietal cortex. We further hypothesized that the mediodorsal thalamus would exhibit altered RSFC in PTSD.

## 2 Methods

Resting-state fMRI scans were shared by independent labs with the ENIGMA PTSD working group for secondary data analysis. Due to the small volumes of the regions-of-interest (ROIs), only high-resolution fMRI data with voxel volumes < 3 mm isotropic voxels were included to balance spatial localization with sufficient statistical power resulting in 397 participants from six sites. Exclusion and inclusion criteria for each site are reported in Appendix I of our supplementary material, and the functional and anatomical scanning parameters for each site are reported in Appendix *II*. All study procedures were approved by local institutional review boards (IRB), and all participants provided written informed consent. Secondary data analysis was deemed exempt by the Duke University Medical Center IRB.

### 2.1 Data processing

**Fig. 1:**
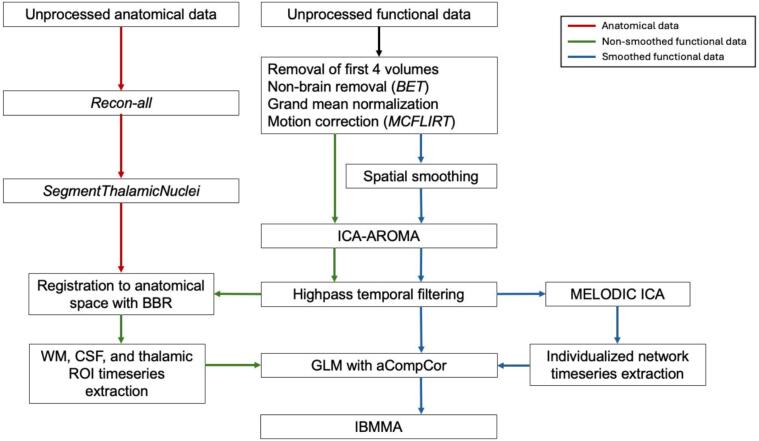
Processing and analysis procedure. Red arrows represent processing of anatomical data, green arrows represent non-smoothed functional data, and blue arrows represent smoothed functional data. Abbreviations: Brain Extraction Tool (BET), boundary-based registration (BBR), cerebrospinal fluid (CSF), generalized linear model (GLM), Image-Based Meta- and Mega-Analysis (IBMMA) (Steele et al., 2025b), ICA-based Automatic Removal of Motion Artifacts (ICA-AROMA) (Pruim et al., 2015), Multivariate Exploratory Linear Optimized Decomposition into Independent Components (MELODIC) (Beckmann & Smith, 2004), region of interest (ROI), white matter (WM). (For interpretation of the references to colour in this figure legend, the reader is referred to the web version of this article.)

**Fig. 2:**
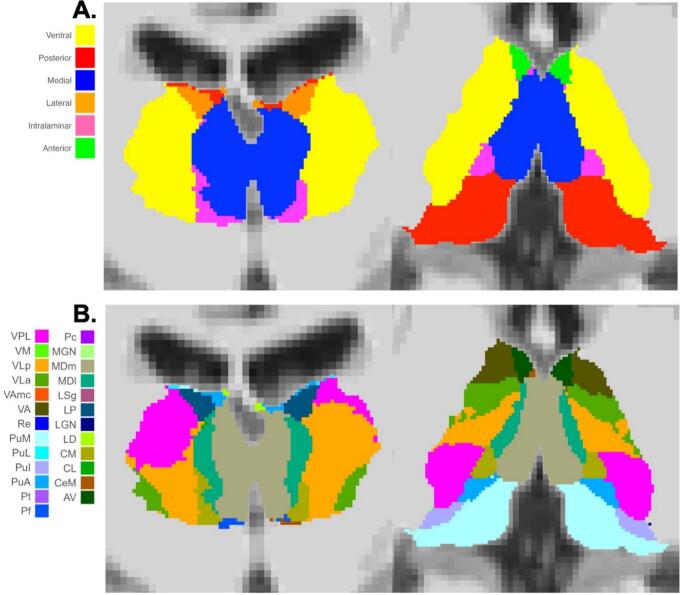
*Example thalamic segmentation visualized in coronal (left) and axial (right) slices at two levels of anatomical granularity.****(A)****Segmentation of the thalamus into six broad subregions: ventral, posterior, medial, lateral, intralaminar, and anterior.****(B)****Finer-grained segmentation of individual thalamic nuclei, based on probabilistic anatomical labeling. Note that not all nuclei are visible in the selected slices due to their limited spatial extent or slice positioning. Abbreviations: anterior pulvinar (PuA), anteroventral (AV), central lateral (CL), central medial (CeM), centromedian (CM), inferior pulvinar (PuI), lateral dorsal (LD), lateral geniculate nucleus (LGN), lateral posterior (LP), lateral pulvinar (PuL), limitans-suprageniculate (LSg), medial geniculate nucleus (MGN), medial pulvinar (PuM), mediodorsal lateral (MDl), mediodorsal medial (MDm), paracentral (Pc), parafascicular (Pf), paratenial (Pt), reuniens (Re), ventral anterior (VA), ventral anterior magnocellular (VAmc), ventral lateral anterior (VLa), ventral lateral posterior (VLp), ventral medial (VM), ventral posterolateral (VPL)*.

Our preprocessing pipeline was developed to maximize the spatial alignment between participant-specific anatomically defined thalamic subregions/nuclei and the functional signal extracted from each region, which was intended to enhance accuracy in capturing region-specific thalamic activity. Fig. 1 outlines the processing and analysis steps. Details of these processing steps can be found in Appendix *IV*. For each participant, we segmented the thalamus into 25 nuclei per hemisphere as shown in Fig. 2 using the probabilistic thalamus atlas within FreeSurfer (Iglesias et al., 2018). Nuclei within the same anatomical subregion were combined to create six thalamic subregions per hemisphere, including the medial (MDm, MDl, Pt, and Re), intralaminar (CL, CeM, CM, Pf, and Pc), lateral (LP and LD), posterior (LGN, MGN, LSg, PuI, PuL, PuA, and PuM), ventral (VPL, VA, VAmc, VLa, VLp, and VM), and anterior (AV) subregions. Thalamic segmentations were visually inspected [NS] to ensure segmentation quality.

### 2.2 Seed to whole brain analysis

Thalamic RSFC was assessed at three levels of granularity: (1) left and right whole thalamus, (2) six thalamic subregions per hemisphere, and (3) 17 thalamic nuclei per hemisphere (Fig. 2). Eight of the 25 nuclei were excluded from analysis due to their small volumes (see *Appendix IV* for details). The timeseries of each thalamic region was entered as a regressor in separate GLMs in the FMRIB Software Library (FSL) (Jenkinson et al., 2012) to create whole brain RSFC maps for each thalamic region while controlling for nuisance signals using aCompCor (Behzadi et al., 2007). Resulting *Z*-statistic maps were normalized to MNI space and resampled to 2 mm isotropic voxels. Normalized *Z*-statistic maps were compared between groups using Image-Based Meta- and Mega-Analysis (IBMMA) software (Steele et al., 2025b). IBMMA performed whole brain voxelwise statistical analysis only using participants with values present for the voxel being tested. IBMMA is well suited for multi-site data where each site may have missing voxel-data at different voxel locations depending on their respective imaging acquisition sequences.

Linear mixed-effects models were used to test for associations between thalamocortical RSFC and PTSD diagnosis while adjusting for sex, age, and age^2^ as fixed effects and site as a random effect (intercept only) (*n* = 397). Associations with PTSD symptom severity were tested using the same modeling approach (*n* = 377). Group-level *Z*-statistic images were enhanced with probabilistic threshold-free cluster enhancement (pTFCE) (Spisak & Heinrichs, 2019) and thresholded at a family-wise error rate (FWER) of *p* < 0.05. Significant clusters comprised of at least 10 contiguous voxels are reported.

Sensitivity analyses were conducted to evaluate the potential influence of confounding variables, including sex, psychotropic medication use, and trauma exposure. To assess the potential impact of sex imbalance between diagnostic groups, a PTSD-by-sex interaction term was examined, and sex-stratified analyses were performed separately in males (*n* = 264) and females (*n* = 157). To assess the impact of psychotropic medication use, a statistical model including medication status as a binary covariate was applied to the subset of participants with available medication data (*n* = 209). Finally, since 29.3 % of our control group was trauma-naive, a sensitivity analysis was performed comparing the PTSD group to trauma-exposed controls (TEC) (*n* = 334). Results of the supplementary analyses were thresholded at *Z* ≥ 3.1 (*p* < 0.001, uncorrected) and compared to the main findings via conjunction analysis to find the spatial intersection of the two contrasts (e.g., PTSD < Controls ∩ PTSD < TEC).

### 2.3 Resting-State networks

Five canonical RSNs were examined, the default mode, salience, central executive, somatosensory, and visual networks. These five networks were chosen for being the most reproducible across six RSN atlases (Doucet et al., 2019). Individualized RSNs were generated for each participant using independent component analysis (ICA) (Fig. S1). Additionally, individualized RSNs were defined using a seed-based functional connectivity approach to replicate the ICA network findings. Compared to traditional approaches using a standard template as a network mask, using individualized networks accounted for participant-specific variability in brain network architecture. Detailed methods for generating individualized networks are provided in Appendix *IV*.

### 2.4 Resting-State network to thalamus analysis

RSFC between individualized RSNs and the bilateral thalamus was calculated for five cortical RSNs. First-level analysis was performed by entering the demeaned individualized-network timeseries into a GLM in FSL while controlling for WM and CSF signals with aCompCor. The resulting RSFC maps were entered into IBMMA for between-group analysis using linear mixed-effects models. Resulting *Z*-statistic maps were masked with the Oxford-Harvard probabilistic thalamus atlas thresholded at 50 %, such that only voxels with at least a 50 % probability of being a part of the thalamus were included. *Z*-statistic maps were thresholded at a FWER of *p* < 0.05. Results were compared with the probabilistic thalamus atlas (Iglesias et al., 2018) to determine which nuclei displayed the greatest overlap with the significant voxels. This procedure was performed on both ICA and seed-based individualized network maps.

## 3 Results

### 3.1 Sample

Demographic and clinical descriptive statistics of our sample are presented in Table 1 and reported by site in Appendix *II*. The proportion of females was significantly higher in the PTSD group compared to the control group (χ^2^ = 25.92; p < 0.001). To assess the potential confounding effects of sex on PTSD-related results, a PTSD-by-sex interaction term was examined for all analyses, and sex-stratified analyses were conducted (Appendix *VI*).

**Table 1:** Demographic and psychiatric measures. T-tests and chi-squared tests were used to evaluate group differences of continuous and discrete variables, respectively. PTSD and MDD severity scores have been normalized between zero and one. Abbreviations: posttraumatic stress disorder (PTSD), major depressive disorder (MDD), standard deviation (SD).

**Variable**	**PTSD** **(n = 182)**	**Controls** **(n = 215)**	**T-statistic or χ^2^**	***p***
Mean Age (SD)	37.16 (11.57)	35.17 (11.30)	t = 1.73	0.084
Female %	51.37 %	26.05 %	χ^2^ = 25.92	<0.001
Trauma Exposed %	100.00 %	70.70 %	χ^2^ = 58.46	<0.001
Mean PTSD Severity (SD)	0.50 (0.13)	0.08 (0.12)	t = 31.48	<0.001
MDD Diagnosis %	69.20 %	0 %	χ^2^ = 210.06	<0.001
Mean MDD Severity (SD)	0.39 (0.17)	0.08 (0.08)	t = 22.04	<0.001

### 3.2 PTSD is associated with altered thalamocortical RSFC

Thalamic RSFC was compared between the PTSD group and controls, with significant results reported in Table 2. No significant RSFC differences of the left or right whole thalamus (low granularity) in PTSD compared to controls (all *p_FWE_* > 0.05).

**Table 2:** Seed-to-whole brain results showing thalamocortical connections with significant RSFC differences in PTSD compared to controls. Bolded regions represent the coarser subregion labels, while non-bolded regions below each subregion label represent nuclei within the subregion. Abbreviations: inferior pulvinar (PuI), lateral geniculate nucleus (LGN), left (L), medial geniculate nucleus (MGN), medial pulvinar (PuM), region-of-interest (ROI), right (R), ventral anterior (VA).

**ROI**	**Size (voxels)**	**Z-Threshold (*p_FWE_* < 0.05)**	**Peak Z-Score**	**X**	**Y**	**Z**	**Peak Voxel Harvard-Oxford Cortical Atlas Label**
**PTSD < Controls**							
**Posterior L.**	849	4.97	6.04	6	12	54	Superior Frontal Gyrus
LGN L.	12	5.03	5.61	−40	4	26	Precentral Gyrus
MGN L.	34	5.06	5.74	−48	34	16	Inferior Frontal Gyrus, pars triangularis
PuI L.	14	5.04	5.65	10	−2	36	Cingulate Gyrus, anterior division
	16	5.04	5.42	48	−2	40	Precentral Gyrus
**Posterior R.**	11	5.03	5.48	8	12	52	Paracingulate Gyrus
PuI R.	1274	4.96	5.75	6	4	62	Supplementary Motor Area
	523	4.96	5.62	4	−44	56	Precuneus Cortex
	36	4.96	5.61	44	12	20	Inferior Frontal Gyrus, pars opercularis
	153	4.96	5.60	12	22	26	Cingulate Gyrus, anterior division
	213	4.96	5.59	32	0	62	Middle Frontal Gyrus
	79	4.96	5.55	62	−36	28	Supramarginal Gyrus, posterior division
	258	4.96	5.53	−8	–32	68	Precentral Gyrus
	54	4.96	5.40	24	−56	40	Lateral Occipital Cortex, superior division
	67	4.96	5.37	40	−44	34	Supramarginal Gyrus, posterior division
	99	4.96	5.32	46	−44	54	Supramarginal Gyrus, posterior division
	62	4.96	5.32	−48	0	38	Precentral Gyrus
	17	4.96	5.24	−34	−54	58	Superior Parietal Lobule
	12	4.96	5.22	26	6	42	Superior Frontal Gyrus
	34	4.96	5.15	−2	−54	64	Precuneus Cortex
	38	4.96	5.14	−42	−46	52	Superior Parietal Lobule
	12	4.96	5.13	48	−42	−2	Middle Temporal Gyrus, temporooccipital part
	15	4.96	5.07	62	−24	44	Supramarginal Gyrus, anterior division
	14	4.96	5.06	−54	–32	34	Supramarginal Gyrus, anterior division
	12	4.96	5.00	54	−30	8	Superior Temporal Gyrus, posterior division
**Ventral L.**	−	−	−	−	−	−	−
VA L.	16	5.04	5.56	−26	−56	36	Superior Parietal Lobule
**PTSD > Controls**							
**Posterior R.**	−	−	−	−	−	−	−
MGN R.	16	5.06	5.83	28	−30	50	Postcentral Gyrus
	39	5.06	5.45	−52	−6	38	Precentral Gyrus
	22	5.06	5.42	60	−6	36	Postcentral Gyrus

Examining thalamic subregions (moderate granularity) revealed that the PTSD group had weaker RSFC between the left posterior thalamus and the pre-supplementary motor area (pre-SMA) in the superior frontal gyrus and weaker RSFC between the right posterior thalamus and the paracingulate gyrus extending ventrally into the dorsal anterior cingulate cortex (dACC). No significant results were found for the other five thalamic subregions (all *p_FWE_* > 0.05).

Examining the thalamus at its component nuclei level (high granularity) revealed group differences in RSFC that went undetected at the coarser subregion-level analysis. Within the posterior subregion, compared to controls, the PTSD group exhibited weaker RSFC between the PuI nucleus and frontal sensorimotor regions (SMA, precentral gyrus), the PuI nucleus and salience regions (dACC), as well as the PuI nucleus and parietal regions (precuneus, supramarginal gyrus, and superior parietal lobule). The PTSD group exhibited weaker RSFC between the left MGN and the inferior frontal gyrus and between the left LGN and the precentral gyrus. Stronger RSFC was found in the PTSD group between the right MGN and the pre/postcentral gyri.

Within the ventral subregion, the PTSD group exhibited weaker RSFC between the left VA nucleus and the superior parietal lobule compared to controls. Visualizations of the main findings are provided in Appendix *IX* (Fig. S2).

### 3.3 PTSD severity is correlated with thalamocortical RSFC

No significant RSFC associations with PTSD symptom severity were found for the left or right whole thalamus (all *p_FWE_* > 0.05). Examining thalamic subregions revealed that PTSD severity was significantly correlated with weaker RSFC between the posterior thalamus (left and right) and the SMA (Table 3). PTSD severity was also significantly correlated with weaker RSFC between the medial thalamus (left and right) and the lateral occipital cortex, as well as between the right medial thalamus and the SMA.

**Table 3:** Seed-to-whole brain results showing thalamocortical connections with significant associations with PTSD severity. Bolded regions represent the coarser subregion labels, while non-bolded regions below each subregion label represent nuclei within the subregion. Abbreviations: anterior pulvinar (PuA), inferior pulvinar (PuI), lateral geniculate nucleus (LGN), left (L), mediodorsal lateral (MDl), mediodorsal medial (MDm), medial pulvinar (PuM), region-of-interest (ROI), right (R), ventral posterolateral (VPL).

**ROI**	**Size (voxels)**	**Z-Threshold (*p_FWE_* < 0.05)**	**Peak Z-Score**	**X**	**Y**	**Z**	**Peak Voxel Harvard-Oxford Cortical Atlas Label**
**Posterior L.**	261	−5.02	−5.78	6	6	46	Supplementary Motor Area
	147	−5.02	−5.68	4	−6	70	Supplementary Motor Area
LGN L.	35	−5.02	−5.45	−42	4	26	Precentral Gyrus
	21	−5.02	−5.45	−56	12	4	Inferior Frontal Gyrus, pars opercularis
	17	−5.02	−5.35	6	8	30	Cingulate Gyrus, anterior division
	14	−5.02	−5.25	2	6	64	Supplementary Motor Area
	20	−5.02	−5.16	8	14	48	Paracingulate Gyrus
PuM L.	140	−5.04	−5.61	4	−20	72	Precentral Gyrus
	28	−5.04	−5.50	4	−26	62	Precentral Gyrus
	33	−5.04	−5.35	18	–22	68	Precentral Gyrus
**Posterior R.**	23	−5.01	−5.35	4	−12	68	Supplementary Motor Area
PuM R.	90	−4.98	−6.15	24	−90	32	Occipital Pole
	55	−4.98	−5.71	4	−12	68	Supplementary Motor Area
	33	−4.98	−5.17	32	−86	18	Lateral Occipital Cortex, superior division
PuA R.	18	−5.08	−5.81	36	−90	16	Occipital Pole
PuI R.	3214	−4.96	−6.37	−28	−8	46	Precentral Gyrus
	644	−4.96	−6.12	26	6	42	Superior Frontal Gyrus
	1788	−4.96	−5.87	10	10	52	Paracingulate Gyrus
	1501	−4.96	−5.71	18	−54	56	Superior Parietal Lobule
	36	−4.96	−5.69	0	−68	−6	Lingual Gyrus
	373	−4.96	−5.55	−14	−54	74	Superior Parietal Lobule
	265	−4.96	−5.53	36	2	−2	Insular Cortex
	55	−4.96	−5.53	–32	10	2	Insular Cortex
	296	−4.96	−5.43	64	–32	28	Supramarginal Gyrus, anterior division
	292	−4.96	−5.34	34	–32	44	Postcentral Gyrus
	12	−4.96	−5.21	−48	−2	4	Central Opercular Cortex
	51	−4.96	−5.19	56	12	14	Inferior Frontal Gyrus, pars opercularis
	73	−4.96	−5.07	50	−2	38	Precentral Gyrus
	68	−4.96	−5.06	44	−14	40	Precentral Gyrus
**Medial L.**	12	−5.03	−5.29	30	−88	18	Lateral Occipital Cortex, superior division
MDm L.	150	−5.01	−5.83	30	−90	18	Occipital Pole
	12	−5.01	−5.17	−8	−90	14	Occipital Pole
**Medial R.**	16	−4.96	−5.27	48	−72	−2	Lateral Occipital Cortex, inferior division
	32	−4.96	−5.22	2	2	62	Supplementary Motor Area
MDm R.	23	−4.97	−5.62	42	−72	22	Lateral Occipital Cortex, superior division
	199	−4.97	−5.52	−12	–22	62	Precentral Gyrus
	41	−4.97	−5.39	10	−20	66	Precentral Gyrus
	28	−4.97	−5.34	6	4	60	Supplementary Motor Area
	29	−4.97	−5.24	0	−46	58	Precuneus Cortex
	29	−4.97	−5.23	46	−74	−2	Lateral Occipital Cortex, inferior division
MDl R.	139	−4.98	−5.97	54	−2	48	Precentral Gyrus
	30	−4.98	−5.74	44	−24	32	Supramarginal Gyrus, anterior division
	32	−4.98	−5.47	46	−38	62	Postcentral Gyrus
	16	−4.98	−5.33	48	−70	−2	Lateral Occipital Cortex, inferior division
	22	−4.98	−5.21	30	−80	−12	Occipital Fusiform Gyrus
	19	−4.98	−5.19	40	−4	58	Precentral Gyrus
	20	−4.98	−5.12	44	–32	46	Postcentral Gyrus
**Ventral L.**	−	−	−	−	−	−	−
VPL L.	16	−5.06	−5.33	8	10	32	Cingulate Gyrus, anterior division

Examining the constituent nuclei of each subregion revealed additional associations. In the posterior subregion, PTSD severity correlated with several thalamic nuclei including LGN, PuM, PuA, and PuI having weaker RSFC with both frontal sensorimotor and occipital regions. In the medial subregion, PTSD severity was correlated with weaker RSFC between the MD nucleus (both MDm and MDl) and cortical regions including precentral gyrus, SMA, and occipital regions. In the ventral subregion, PTSD severity significantly correlated with weaker RSFC between the left VPL nucleus and the dACC. Visualizations of the main findings are provided in Appendix *IX* (Fig. S3).

### 3.4 Analysis of confounds

To assess potential confounds, we conducted analyses adjusting for sex (Appendix *VI*), psychotropic medication use (Appendix *VII*), and trauma exposure (Appendix *VIII*). No significant results were found for the interaction of PTSD and sex (all *p_FWE_* > 0.05), and sex-stratified analyses found significant associations in the medial, posterior, and ventral subregions for males, while female participants showed significant associations in the posterior subregion only. Adjusting for psychotropic medication use provided consistent results, as significant associations were found in the medial, posterior, and ventral subregions of the thalamus. Comparing the PTSD group to the TEC group provided consistent results, as associations in the medial, posterior, and ventral subregions remained significant.

### 3.5 PTSD is associated with stronger thalamic RSFC to resting-state networks

**Fig. 3:**
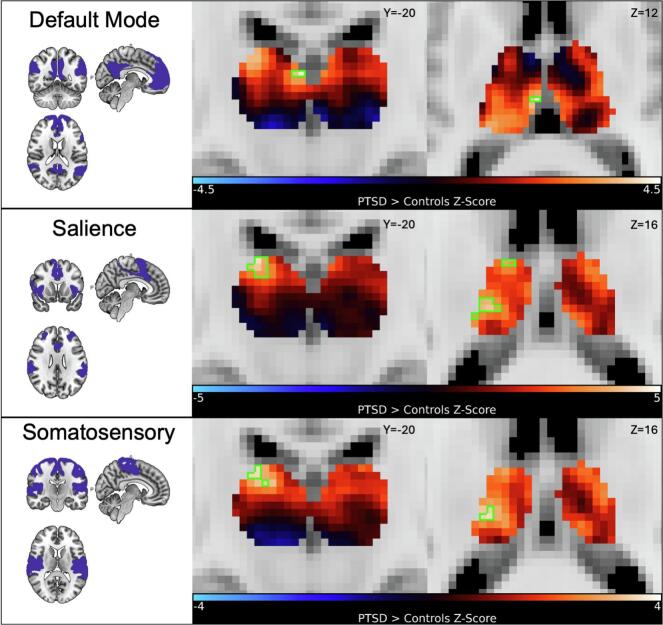
*Coronal (left) and axial (right) views of thalamic RSFC between RSNs and the bilateral thalamus by contrasting cases > controls. Green boxes outline voxels that displayed significantly stronger RSFC between the default (top), salience (middle), and somatosensory (bottom) RSNs and thalamus in PTSD compared to control participants (p_FWE_ < 0.05).* (For interpretation of the references to colour in this figure legend, the reader is referred to the web version of this article.)

RSFC between individualized RSNs and every voxel within the thalamus was calculated. PTSD participants exhibited stronger RSFC between the default mode network and a voxel cluster that localized to the left MDm nucleus of the thalamus (Fig. 3) (max-*Z* = 4.19, *p_FWE_* = 0.007, peak-MNI = [-2, −20, 12]).

PTSD participants exhibited stronger RSFC between the salience network and voxel clusters in somatomotor regions of the thalamus. These clusters localized to the left VPL (max-*Z* = 4.54; *p_FWE_* < 0.001, peak-MNI = [-14, −20, 18]) and left VLa (max-*Z* = 3.96; *p_FWE_* = 0.010, peak-MNI = [-10, −8, 16]) nuclei.

PTSD participants exhibited stronger RSFC between the somatosensory network and a voxel cluster in the left VPL nucleus (max-*Z* = 3.73; *p_FWE_* = 0.016), the major somatosensory processing region of the thalamus.

No significant associations with PTSD were found for the central executive network or visual network (all *p_FWE_* > 0.05).

### 3.6 Replication of results

We tested the robustness of these results by using an alternate method to define RSNs with a seed-based FC approach. Stronger RSFC in PTSD compared to controls was reproduced between the seed-based definition of the default mode network and voxels within the MDm nucleus (max-*Z* = 2.98; *p* = 0.003). Stronger RSFC in PTSD compared to controls between the salience network and voxels within the VPL (max-*Z* = 4.22; *p* < 0.001) and VLa (max-*Z* = 2.97; *p* = 0.003) nuclei was successfully replicated, as was stronger RSFC between the somatosensory network and voxels within the VPL nucleus (max-*Z* = 3.90; *p* < 0.001). Consistent findings across distinct methodological approaches underscore the robustness of these results.

## 4 Discussion

The thalamus is a critical brain structure composed of a heterogeneous set of nuclei that mediate crucial sensorimotor, affective, and cognitive brain functions. We sought to understand how thalamic function is impacted by PTSD by measuring RSFC of thalamic subregions and nuclei with cortical structures and networks. We found the pulvinar and mediodorsal thalamic nuclei exhibited weaker RSFC with sensorimotor and salience regions, while the MGN exhibited stronger RSFC with the sensorimotor cortex in PTSD. Whole-thalamus RSFC analysis revealed no significant associations with PTSD, highlighting subregion-specific alterations. At the network level, the PTSD group showed stronger connectivity between localized thalamic regions and key intrinsic brain networks, including the default mode, salience, and somatosensory networks. The default mode network of PTSD participants had stronger RSFC with the mediodorsal thalamus, while the salience and somatosensory networks exhibited stronger RSFC with somatomotor thalamic nuclei.

### 4.1 Altered connectivity of posterior subregion nuclei in PTSD

Our findings reveal a dissociation in RSFC of posterior thalamic nuclei in PTSD, with the pulvinar exhibiting widespread hypoconnectivity and the MGN displaying hyperconnectivity. Specifically, the PTSD group showed weaker connectivity between the pulvinar and cortical regions supporting voluntary motor planning (pre-SMA, SMA), primary motor/somatosensory processing (precentral gyrus, postcentral gyrus, superior parietal lobule), and salience processing (dACC, insula). By contrast, the MGN exhibited stronger connectivity in PTSD with primary sensorimotor regions, namely the precentral and postcentral gyri, suggesting enhanced engagement of auditory-sensorimotor circuits in PTSD. These findings replicate prior research that reported weaker RSFC between the pulvinar and the precentral gyrus, postcentral gyrus, supramarginal gyrus, precuneus, and superior parietal lobule in PTSD (Terpou et al., 2018). At a coarser resolution, prior research reported weaker whole thalamic RSFC with the dACC (Yin et al., 2011) and mixed connectivity patterns with the sensorimotor cortex (Jeon et al., 2020).

A possible explanation of this dissociation within the posterior thalamus is that trauma sensitizes the auditory-sensorimotor loop (driving hyperarousal) while disrupting thalamocortical circuits involved in voluntary control and cognitive regulation. This explanation is supported by prior work that has found consistently reduced activation of the precentral gyrus and medial frontal cortex (Patel et al., 2012) alongside weaker precentral-cingulate (Kennis et al., 2014) and stronger precentral-autonomic network coupling in PTSD (Thome et al., 2016). Further supporting this, Terpou et al. (2018) proposed that weaker pulvinar-precentral connectivity in PTSD reflects diminished voluntary motor control, while increased precentral-autonomic coupling supports enhanced involuntary, subcortically driven responses. The pre-SMA facilitates the initiation and inhibition of voluntary actions (Wolpe et al., 2022) while both pulvinar-preSMA and pulvinar-occipital coactivations are associated with higher-order cognitive processes (Barron et al., 2015), such as the voluntary planning of actions. Thus, diminished pulvinar connectivity with these regions in PTSD may indicate reduced capacity for voluntary sensorimotor regulation and context-sensitive behavior.

By contrast, increased RSFC between the MGN and sensorimotor cortex may reflect heightened subcortical responsiveness to trauma-related auditory stimuli. The MGN is a primary relay in the auditory thalamus and plays a central role in fear conditioning and associative learning through its reciprocal connections with limbic structures, including the amygdala (Weinberger, 2011). We hypothesize that stronger MGN-sensorimotor coupling reflects an overactive sensory-autonomic pathway supporting stimulus-driven motor responses that are biased toward threat.

### 4.2 Altered connectivity of medial subregion nuclei in PTSD

The mediodorsal thalamus interacts with the amygdala and medial prefrontal cortex to consolidate and extinguish fear memories (Lee & Shin, 2016), and is crucial for emotion, learning and memory. Engagement of the mediodorsal thalamus during internally focused cognition drives excitatory activation of the default mode network, which is believed to increase cortical synchrony and thereby support the maintenance of mental representations (Harrison et al., 2022). The mediodorsal thalamus displayed stronger connectivity with the default mode network in PTSD and weaker connectivity with sensory or motor regions in the frontal, parietal, and occipital lobes with increasing PTSD severity. Our findings show that PTSD is associated with enhanced recruitment of the mediodorsal thalamus into the default mode network and concurrent weakening of mediodorsal connectivity to sensorimotor regions.

Together, this pattern may indicate that in PTSD, internal mnemonic and emotional processes exert a stronger influence on the default mode network and mediodorsal thalamus than external sensory inputs. These findings provide support for the hypothesis espoused by Chaposhloo and colleagues (2023) that the precuneus, a central node of the default mode network, exhibits a shift from a sensory-driven to an affective memory-based representation scheme in PTSD driven by weaker pulvinar-precuneus connectivity (Terpou et al., 2018) and stronger anterior hippocampus-precuneus connectivity (Chaposhloo et al., 2023). We hypothesize that this shift in thalamocortical dynamics could explain the cognitive symptoms of the disorder and contribute to dysfunctional fear extinction in PTSD. Future research could test this hypothesis by examining the content of default mode network representations through multivoxel pattern analysis during tasks that contrast externally driven sensory processing with internally generated affective memory retrieval and assess whether a shift towards memory-based representations correlates with more severe cognitive symptoms and impaired fear extinction in PTSD.

### 4.3 Altered connectivity of ventral subregion nuclei in PTSD

The VPL nucleus, which includes the ventral posteromedial nucleus, acts as the major somatosensory processing nucleus of the thalamus (Song & Semework, 2015), while the VA and VLa nuclei are motor output nuclei (Sieveritz et al., 2019). Greater PTSD symptom severity correlated with weaker connectivity between the VPL nucleus and a dACC substructure associated with pain processing (MacIver et al., 2008), and the PTSD group showed weaker RSFC between the VA nucleus and the superior parietal lobule. Furthermore, we found stronger connectivity between the salience and somatosensory networks with part of the VPL nucleus, and between the salience network and part of the VLa nucleus.

Heightened and diminished somatosensory sensitivity, altered motor responding to tactile input, and tonic immobility during recall of traumatic memories are characteristic PTSD symptoms attributed to somatosensory and salience network processing (Kearney & Lanius, 2022). It is hypothesized that disruptions in subcortico-cortical connections may be responsible for the various somatic symptoms experienced post-trauma. As such, top-down overmodulation of subcortical somatosensory hubs by the cortex may lead to dissociative symptoms, while hyperconnectivity from the subcortex to the cortex may lead to heightened arousal due to an exaggerated influence of subcortical sensory processing hubs on cortical processing. Here, we show that weaker connectivity between the VPL and VA nuclei with the dACC and superior parietal lobule, respectively, alongside stronger connectivity of the VPL and VLa nuclei with the salience and somatosensory networks, may be an important link in these processes. Although data on dissociative symptoms were not available, future research should examine thalamocortical connectivity differences between dissociative and non-dissociative subtypes of PTSD to clarify the contributions to distinct symptom presentations.

### 4.4 Limitations and Strengths

Several limitations must be considered when interpreting our results. The PTSD and control groups were imbalanced with regard to sex. We addressed this by testing the interaction of PTSD and sex and conducted sex-stratified analyses, both of which yielded consistent findings. Additionally, the use of psychotropic medications is a common confound in psychiatric research. Our supplementary analysis that adjusted for psychotropic medication produced consistent results. However, the specific classes of psychotropic medications being used were not available.

A major strength was the use of high spatial-resolution fMRI to examine distinct thalamic subregions and nuclei and mitigate partial volume effects. Considering individual differences in thalamic nuclei structure and network architecture by individually parcellating the thalamus and RSNs for each participant was another strength.

## 5 Conclusions

Coarse parcellation of the functionally diverse thalamus has limited our understanding of its specific role in PTSD. We found PTSD was associated with weaker RSFC of posterior and medial thalamic nuclei with sensorimotor and salience regions. Stronger connectivity of localized thalamic regions with the default mode, salience, and somatosensory networks was observed in PTSD. Fine-grained thalamic mapping is important for uncovering network-level disruptions that are linked to sensory dysregulation and salience processing abnormalities in PTSD.

## CRediT authorship contribution statement

**Nick Steele:** Writing – review & editing, Writing – original draft, Methodology, Formal analysis, Conceptualization. **Ahmed Hussain:** Writing – review & editing, Resources, Data curation. **Delin Sun:** Writing – review & editing, Supervision, Resources. **Courtney Russell:** Writing – review & editing, Resources, Data curation. **Ashley A. Huggins:** Writing – review & editing, Data curation. **Nicholas D. Davenport:** Writing – review & editing, Data curation. **Seth G. Disner:** Writing – review & editing, Data curation. **Scott R. Sponheim:** Writing – review & editing, Data curation. **Thomas Straube:** Writing – review & editing, Data curation. **David Hofmann:** Writing – review & editing, Data curation. **Shmuel Lissek:** Writing – review & editing, Data curation. **Hannah Berg:** Writing – review & editing, Data curation. **Daniel W. Grupe:** Writing – review & editing, Data curation. **Jack B. Nitschke:** Writing – review & editing, Data curation. **Richard J. Davidson:** Writing – review & editing, Data curation. **Ruth Lanius:** Writing – review & editing, Data curation. **Maria Densmore:** Writing – review & editing, Data curation. **Jean Théberge:** Writing – review & editing, Data curation. **Richard W.J. Neufeld:** Writing – review & editing, Data curation. **Sophia I. Thomopoulos:** Writing – review & editing, Resources. **Paul M. Thompson:** Writing – review & editing, Resources. **Rajendra A. Morey:** Writing – review & editing, Supervision, Resources, Data curation.

## Data Availability

The authors do not have permission to share data.

## References

[b0005] American Psychiatric Association. (2013). Diagnostic and statistical manual of mental disorders (5th ed.). https://doi.org/10.1176/appi.books.9780890425596.

[b0010] Barron D.S., Eickhoff S.B., Clos M., Fox P.T. (2015). Human pulvinar functional organization and connectivity. Hum. Brain Mapp..

[b0015] Beckmann C.F., Smith S.M. (2004). Probabilistic independent component analysis for functional magnetic resonance imaging. IEEE Trans. Med. Imaging.

[b0020] Behzadi Y., Restom K., Liau J., Liu T.T. (2007). A Component based Noise Correction Method (CompCor) for BOLD and Perfusion based fMRI. Neuroimage.

[b0025] Cabrera-Álvarez J., Doorn N., Maestú F., Susi G. (2023). Modeling the role of the thalamus in resting-state functional connectivity: Nature or structure. PLoS Comput. Biol..

[b0030] Chaposhloo M., Nicholson A.A., Becker S., McKinnon M.C., Lanius R., Shaw S.B. (2023). Altered Resting-State functional connectivity in the anterior and posterior hippocampus in Post-traumatic stress disorder: the central role of the anterior hippocampus. NeuroImage : Clinical.

[b0035] Cortes N., Ladret H.J., Abbas-Farishta R., Casanova C. (2024). The pulvinar as a hub of visual processing and cortical integration. Trends Neurosci..

[b0040] Doucet G.E., Lee W.H., Frangou S. (2019). Evaluation of the spatial variability in the major resting‐state networks across human brain functional atlases. Hum. Brain Mapp..

[b0045] Fratzl A., Koltchev A.M., Vissers N., Tan Y.L., Marques-Smith A., Stempel A.V., Branco T., Hofer S.B. (2021). Flexible inhibitory control of visually evoked defensive behavior by the ventral lateral geniculate nucleus. Neuron.

[b0050] Gu H., Zhao F., Liu Z., Cao P. (2025). Defense or death? a review of the neural mechanisms underlying sensory modality-triggered innate defensive behaviors. Curr. Opin. Neurobiol..

[b0055] Guillery R.W. (1995). Anatomical evidence concerning the role of the thalamus in corticocortical communication: a brief review. J. Anat..

[b0060] Harrison B.J., Davey C.G., Savage H.S., Jamieson A.J., Leonards C.A., Moffat B.A., Glarin R.K., Steward T. (2022). Dynamic subcortical modulators of human default mode network function. Cereb. Cortex.

[b0065] Hwang K., Bertolero M.A., Liu W.B., D’Esposito M. (2017). The Human Thalamus is an Integrative Hub for Functional Brain Networks. J. Neurosci..

[b0070] Hwang W.J., Kwak Y.B., Cho K.I.K., Lee T.Y., Oh H., Ha M., Kim M., Kwon J.S. (2022). Thalamic Connectivity System across Psychiatric Disorders: Current Status and Clinical Implications. Biological Psychiatry Global Open Science.

[b0075] Iglesias J.E., Insausti R., Lerma-Usabiaga G., Bocchetta M., Van Leemput K., Greve D.N., van der Kouwe A., Fischl B., Caballero-Gaudes C., Paz-Alonso P.M. (2018). A probabilistic atlas of the human thalamic nuclei combining ex vivo MRI and histology. Neuroimage.

[b0080] Jenkinson M., Beckmann C.F., Behrens T.E.J., Woolrich M.W., Smith S.M. (2012). FSL. Neuroimage.

[b0085] Jeon S., Lee Y.J., Park I., Kim N., Kim S., Jun J.Y., Yoo S.Y., Lee S.H., Kim S.J. (2020). Resting State Functional Connectivity of the Thalamus in North Korean Refugees with and without Posttraumatic stress Disorder. Sci. Rep..

[b0090] Kawabata, K., Bagarinao, E., Watanabe, H., Maesawa, S., Mori, D., Hara, K., Ohdake, R., Masuda, M., Ogura, A., Kato, T., Koyama, S., Katsuno, M., Wakabayashi, T., Kuzuya, M., Hoshiyama, M., Isoda, H., Naganawa, S., Ozaki, N., & Sobue, G. (2021). Bridging large-scale cortical networks: Integrative and function-specific hubs in the thalamus. *iScience*, *24*(10). https://doi.org/10.1016/j.isci.2021.103106. 10.1016/j.isci.2021.103106 PMC8479782 34622159

[b0095] Kearney B.E., Lanius R.A. (2022). The brain-body disconnect: a somatic sensory basis for trauma-related disorders. Front. Neurosci..

[b0100] Kennis M., Rademaker A.R., van Rooij S.J.H., Kahn R.S., Geuze E. (2014). Resting state functional connectivity of the anterior cingulate cortex in veterans with and without post‐traumatic stress disorder. Hum. Brain Mapp..

[b0105] Kim S.J., Lyoo I.K., Lee Y.S., Kim J., Sim M.E., Bae S.J., Kim H.J., Lee J.-Y., Jeong D.-U. (2007). Decreased cerebral blood flow of thalamus in PTSD patients as a strategy to reduce re-experience symptoms. Acta Psychiatr. Scand..

[b0110] Koch S.B.J., van Zuiden M., Nawijn L., Frijling J.L., Veltman D.J., Olff M. (2016). Aberrant Resting-State Brain activity in Posttraumatic stress Disorder: a Meta-Analysis and Systematic Review. Depress. Anxiety.

[b0115] Lee S., Shin H.-S. (2016). The role of mediodorsal thalamic nucleus in fear extinction. J. Anal. Sci. Technol..

[b0120] Li K., Fan L., Cui Y., Wei X., He Y., Yang J., Lu Y., Li W., Shi W., Cao L., Cheng L., Li A., You B., Jiang T. (2022). The human mediodorsal thalamus: Organization, connectivity, and function. Neuroimage.

[b0125] MacIver K., Lloyd D.M., Kelly S., Roberts N., Nurmikko T. (2008). Phantom limb pain, cortical reorganization and the therapeutic effect of mental imagery. Brain.

[b0130] Morey R.A., Dunsmoor J.E., Haswell C.C., Brown V.M., Vora A., Weiner J., Stjepanovic D., Wagner H.R., LaBar K.S. (2015). Fear learning circuitry is biased toward generalization of fear associations in posttraumatic stress disorder. Transl. Psychiatry.

[b0135] Morey R.A., Petty C.M., Cooper D.A., LaBar K.S., McCarthy G. (2008). Neural systems for executive and emotional processing are modulated by symptoms of posttraumatic stress disorder in Iraq War veterans. Psychiatry Res..

[b0140] Patel R., Spreng R.N., Shin L.M., Girard T.A. (2012). Neurocircuitry models of posttraumatic stress disorder and beyond: a meta-analysis of functional neuroimaging studies. Neurosci. Biobehav. Rev..

[b0145] Pruim R.H.R., Mennes M., van Rooij D., Llera A., Buitelaar J.K., Beckmann C.F. (2015). ICA-AROMA: a robust ICA-based strategy for removing motion artifacts from fMRI data. Neuroimage.

[b0150] Sherman S.M. (2016). Thalamus plays a central role in ongoing cortical functioning. Nat. Neurosci..

[b0155] Shine J.M. (2021). The thalamus integrates the macrosystems of the brain to facilitate complex, adaptive brain network dynamics. Prog. Neurobiol..

[b0160] Sieveritz B., García-Muñoz M., Arbuthnott G.W. (2019). Thalamic afferents to prefrontal cortices from ventral motor nuclei in decision-making. Eur. J. Neurosci..

[b0165] Sommer M.A. (2003). The role of the thalamus in motor control. Curr. Opin. Neurobiol..

[b0170] Song W., Semework M. (2015). Tactile representation in somatosensory thalamus (VPL) and cortex (S1) of awake primate and the plasticity induced by VPL neuroprosthetic stimulation. Brain Res..

[b0175] Spisak, T., & Heinrichs, H. (2019). pTFCE: Probabilistic Threshold-Free Cluster Enhancement of Neuroimages [Computer software]. https://spisakt.github.io/pTFCE.

[b0180] Steele N., Hussain A., Baird C.L., Haswell C.C., Sun D., Rangel-Jimenez L., Abdallah C.G., Angstadt M., May G., Berg H., Blackford J.U., Cisler J.M., Daniels J.K., Davenport N.D., Davidson R.J., Densmore M., Disner S.G., El-Hage W., Etkin A., Morey R.A. (2025). Volumetric differences of thalamic nuclei are associated with post-trauma psychopathology. Biol. Psychiatry: Cognit. Neurosci. Neuroimaging.

[b0185] Steele N., Huggins A.A., Morey R.A., Hussain A., Russell C., Suarez-Jimenez B., Pozzi E., Jameei H., Schmaal L., Veer I.M., Waller L., Jahanshad N., Thomopoulos S.I., Salminen L.E., Olff M., Frijling J.L., Veltman D.J., Koch S.B.J., Nawijn L., Sun D. (2025). Image-based meta- and mega-analysis (IBMMA): a unified framework for large-scale, multi-site, neuroimaging data analysis. Neuroimage.

[bib216] Steele N., Hussain A., Sun D., Baird C.L., Russell C., Jahanshad N., Salminen L., Olff M., Frijling J.L., Veltman D.J., Koch S.B.J., Nawijn L., van Zuiden M., Wang L., Zhu Y., Li G., Stein D.J., Ipser J., Koopowitz S., Neria Y., Zhu X., Ravid O., Zilcha-Mano S., Lazarov A., Suarez-Jimenez B., Huggins A.A., Stevens J., Ressler K., Jovanovic T., van Rooij S.J.H., Fani N., Dennis E.L., Tate D.F., Cifu D.X., Walker W.C., Wilde E.A., Rektor I., Říha P., Kaufman M.L., Lebois L.A.M., Baker J.T., King A., Liberzon I., Angstadt M., Davenport N.D., Disner S.G., Sponheim S.R., Straube T., Hofmann D., Lu G.M., Qi R., Etkin A., Maron-Katz A., Wang X., Kunch A., Xie H., Quidé Y., El-Hage W., Lissek S., Berg H., Bruce S.E., Cisler J., Ross M., Herringa R., Grupe D.W., Nitschke J.B., Davidson R.J., Larson C., deRoon-Cassini T.A., Tomas C.W., Fitzgerald J.M., Feola B., Blackford J.U., Olatunji B.O., May G., Nelson S.M., Gordon E.M., Abdallah C.G., Lanius R., Densmore M., Théberge J., Neufeld R.W.J., Thompson P.M., Morey R.A. (2025). Disrupted Intra-Thalamic and Thalamo-Cortical Structural Covariance Networks in Posttraumatic Stress Disorder. Network Neurosci..

[b0190] Terpou B.A., Densmore M., Théberge J., Frewen P., McKinnon M.C., Lanius R.A. (2018). Resting-state pulvinar-posterior parietal decoupling in PTSD and its dissociative subtype. Hum. Brain Mapp..

[b0195] Thome J., Densmore M., Frewen P.A., McKinnon M.C., Théberge J., Nicholson A.A., Koenig J., Thayer J.F., Lanius R.A. (2016). Desynchronization of autonomic response and central autonomic network connectivity in posttraumatic stress disorder. Hum. Brain Mapp..

[b0200] Weinberger N.M. (2011). The medial geniculate, not the amygdala, as the root of auditory fear conditioning. Hear. Res..

[b0205] Wolpe N., Hezemans F.H., Rae C.L., Zhang J., Rowe J.B. (2022). The pre-supplementary motor area achieves inhibitory control by modulating response thresholds. Cortex.

[b0210] Yin Y., Jin C., Hu X., Duan L., Li Z., Song M., Chen H., Feng B., Jiang T., Jin H., Wong C., Gong Q., Li L. (2011). Altered resting-state functional connectivity of thalamus in earthquake-induced posttraumatic stress disorder: a functional magnetic resonance imaging study. Brain Res..

[b0215] Zhang Y., Chen H., Long Z., Cui Q., Chen H. (2016). Altered effective connectivity network of the thalamus in post-traumatic stress disorder: a resting-state FMRI study with granger causality method. Applied Informatics.

