# Supplementary material for: Disrupted thalamocortical functional connectivity and canonical resting-state network integration in posttraumatic stress disorder
Source: Neuroimage Clin. 2025 Dec 12;49:103927. doi: 10.1016/j.nicl.2025.103927 (PMC12775856; doi:10.1016/j.nicl.2025.103927)
Supplement: Supplementary Data 1 [file mmc1.docx]

**Supplementary Materials**

**Table of Contents**

***STUDY SITE INFORMATION***

**Appendix I - Inclusion and exclusion criteria.............................................................................2**

**Appendix II - Scanning parameters.............................................................................................4**

**Appendix III - Demographic and clinical data...........................................................................6**

***SUPPLEMENTARY METHODS***

**Appendix IV - Supplementary Methods......................................................................................7**

***SUPPLEMENTARY RESULTS***

**Appendix V - Volumetric data....................................................................................................10**

**Appendix VI - Sex-stratified analyses........................................................................................14**

**Appendix VII - Psychotropic medication use analysis.............................................................24**

**Appendix VIII - Trauma-exposed controls comparison..........................................................28**

***SUPPLEMENTARY FIGURES***

**Appendix IX - Thalamocortical RSFC figures..........................................................................33**

***SUPPLEMENTARY REFERENCES***

**Appendix X - Supplementary References..................................................................................35**

**Appendix I: Inclusion and Exclusion Criteria**

| **Site** | **Location** | **Inclusion criteria** | **Exclusion criteria** |
| --- | --- | --- | --- |
| Duke | Durham, NC, USA | Veterans 18-65 years of age, fluent in English, free of implanted metal objects or metal shards in eyes | Axis I psychiatric conditions other than PTSD or MDD; current substance abuse or lifetime substance dependence (other than nicotine); high risk for suicide, claustrophobia; neurological disorders; learning disability or developmental delay; major medical conditions |
| Minneapolis VA | Minneapolis, MN, USA | OEF and/or OIF veteran 22-60 years of age who had been exposed to combat during their deployment(s). | Current psychosis; current DSM-IV substance abuse or dependence other than alcohol, caffeine, or nicotine; moderate or severe traumatic brain injury; neurologic condition other than TBI; current unstable medical condition that would likely affect brain function (e.g., uncontrolled diabetes); significant imminent risk of suicidal or homicidal behavior. |
| University of Minnesota | Minneapolis, MN, USA | Individuals 18-65 years of age with history of combat-related trauma | Current of past history of psychosis, bipolar disorder, delirium, dementia, amnestic disorder, or intellectual disability; suicidality; substance use disorder within past six months; pregnancy; current or past medical illnesses that may confound study results or place participant at risk; current use of any medication that alters central nervous system function including antidepressants, benzodiazepines, anti-psychotics, mood-stabilizers, anti-parkinsonian agents, anti-convulsants, sleep medications, pain medications, and anti-hypertensives; MRI contraindications |
| Münster | Münster, Germany | Individuals who have experienced IPV trauma; right-handed; normal or corrected-to-normal vision | No control had lifetime PTSD; MRI contraindications |
| Western Ontario | London, Ontario, Canada | Primary diagnosis of PTSD for patients | Incompatibilities with scanning conditions, previous neurologic and development illness, comorbid schizophrenia or bipolar disorder, alcohol or substance abuse, a history of head trauma, or pregnancy during scan. participants were excluded if they had implants  or metal that do not comply with 3T fMRI safety standards for research, a history of head injury  with a loss of consciousness, significant untreated medical illness, a history of neurological  disorders, history of any pervasive developmental disorders, pregnancy, and current use of any  psychotropic medication within one month prior to study. PTSD individuals were further  excluded if they reported a history of bipolar disorder, schizophrenia, or substance-use disorder  prior to participation of the study |
| Wisconsin-Grupe | Madison, WI, USA | Adults 18-50 with exposure to 1+ life-threatening war zone trauma events; capable of giving informed consent and fluent in English; clear evidence of war zone trauma exposure in Iraq or Afghanistan since 2001 (e.g., Combat Action Ribbon [Marines], Combat Infantry Badge [Army]); stable pharmacological or psychotherapeutic treatment for at least 8 weeks prior to beginning of study | Weight >352 pounds or over; pregnancy or current breastfeeding; Metallic implants such as prostheses or aneurysm clip, or electronic implants such as cardiac pacemakers; Neurological or serious medical condition; History of seizures or seizure disorder; Moderate or severe traumatic brain injury; Current active substance dependence or dependence within 3 months (other than nicotine); bipolar disorder, schizophrenia, schizoaffective disorder, psychotic disorder NOS, delirium, or any DSM-IV cognitive disorder; Severe psychiatric instability or severe situational life crises, (e.g., suicidality, homicidality); extensive experience in yoga or meditation; Current use of benzodiazepines and beta-blockers |

**Table S1:** Inclusion and exclusion criteria of each site.

**Appendix II: Scanning Parameters**

| **Site** | **Scanner** | **Field strength** | **Head coil channels** | **Sequence** | **Voxel size (mm)** | **FOV (mm)** | **Orientation** | **TR (ms)** | **TE (ms)** | **Flip angle** |
| --- | --- | --- | --- | --- | --- | --- | --- | --- | --- | --- |
| Duke | Philips Ingenia | 3T | NA | Gradient Recalled, segmented k-space | 1.8x1.8x4 | 230x230 | Axial oblique | 2000 | NA | 90 |
| Minnesota VA | Siemens Tim Trio | 3T | 32 | Gradient echo EPI | 2x2x2 | 212x212 | Axial | 1320 | 30 | 90 |
| University of Minnesota | Siemens MAGNETOM Prisma | 3T | 32 | EPI | 2.4x2.4x2.4 | 208x208 | Axial | 1500 | 30.4 | 75 |
| Münster | Siemens MAGNETOM Prisma | 3T | 20 | Gradient-echo planar sequence BOLD | 2.3x2.3x3 | 208x208 | Axial | 2080 | 30 | 90 |
| Western Ontario | Siemens | 3T | 32 | Gradient echo | 2x2x2 | 192x192 | Axial | 3000 | 20 | 90 |
| Wisconsin-Grupe | GE X750 Discovery | 3T | 8 | EPI | 1.719x1.719x4 | 220x220 | Sagittal | 2000 | 20 | 60 |

**Table S2:** Functional scanning parameters for each site.

| **Site** | **Scanner** | **Field strength** | **Head coil channels** | **Sequence** | **Voxel size (mm)** | **FOV (mm)** | **Orientation** | **Repetition time (TR, ms)** | **Echo time (TE, ms)** | **Flip angle** |
| --- | --- | --- | --- | --- | --- | --- | --- | --- | --- | --- |
| Duke | GE Discovery MR750 | 3T | 8 | FSPGR BRAVO | 1x1x1 | 256 | Axial | 8.16 | 3.18 | 12 |
| Minnesota VA | Siemens Tim Trio | 3T | 12 | MPRAGE | 1x1x1 | 256x256 | Coronal | 2530 | 3.7 | 7 |
| University of Minnesota | Siemens Prisma | 3T | 32 | NA | 0.9x0.9x0.9 | NA | NA | NA | NA | NA |
| Münster | Siemens Prisma | 3T | 32 | MPRAGE | 1x1x1 | 256x256 | Sagittal | 2130 | 2.28 | 8 |
| Western Ontario | Siemens Biograph mMR | 3T | 32 | MPRAGE | 1x1x1 | 256x240x192 | Axial | 2300 | 2.98 | 9 |
| Wisconsin-Grupe | GE Discovery X750 | 3T | 8 | MPRAGE | 1x1x1 | 256x256 | Sagittal | 1900 | 2.5 | 9 |

**Table S3:** Anatomical scanning parameters for each site.

**Appendix III: Demographic and Clinical Data**

| **Site** | ***N*** | **% Female** | **Age (*SD*)** | **PTSD %** | **PTSD Severity** | **PTSD Tool** | **MDD %** | **MDD Severity** | **MDD Tool** | **Psychotropic Medication Use %** |
| --- | --- | --- | --- | --- | --- | --- | --- | --- | --- | --- |
| Duke | 34 | 17.65 | 39.85 (12.75) | 26.47 | 0.25 | DTS | 17.65 | 0.16 | BDI | 15.38 |
| Minnesota VA | 90 | 2.22 | 32.57 (7.54) | 24.44 | 0.22 | CAPS-4 | 7.06 | 0.16 | BDI | NA |
| University of Minnesota | 48 | 10.42 | 42.71 (10.02) | 18.75 | 0.12 | CAPS-4 | 8.51 | 0.15 | BDI | NA |
| Münster | 44 | 79.55 | 26.23 (6.83) | 36.36 | NA | SCID-4 | 20.45 | 0.14 | BDI | 56.25 |
| Western Ontario | 159 | 62.26 | 38.83 (12.39) | 72.33 | 0.37 | CAPS-4, CAPS-5 | 54.73 | 0.3 | BDI | 0.00 |
| Wisconsin-Grupe | 22 | 13.64 | 30.41 (6.11) | 50 | 0.26 | CAPS-4 | 36.36 | 0.2 | BDI | 30.43 |
| **Total** | **397** | **30.96** | **35.10 (9.27)** | **38.06** | **0.24** | **-** | **24.13** | **0.19** | **-** | **16.16** |

**Table S4:** Demographic and clinical data broken down by site. Symptom severity scores have been normalized between zero and one. Abbreviations: Beck Depression Inventory (BDI); Clinician-Administered PTSD Scale (CAPS); Davidson Trauma Scale (DTS); major depressive disorder (MDD); posttraumatic stress disorder (PTSD); standard deviation (SD); Structured Clinical Interview for the DSM (SCID); Veterans Affairs (VA).

**Appendix IV: Supplementary Methods**

**Data Processing**

T1-weighted anatomical scans were preprocessed and segmented in FreeSurfer. High-resolution resting-state fMRI scans were preprocessed with the FMRIB Software Library (FSL) (Jenkinson et al., 2012). Initial preprocessing steps included removal of the first four volumes to allow for magnetization stabilization, slice-time correction of single-echo scans, non-brain tissue removal, grand-mean intensity normalization, and motion correction. Participants with absolute head motion greater than 0.5mm were excluded from analyses to minimize the impact of motion artifacts on functional connectivity results (*n* = 26). Following initial preprocessing, two parallel preprocessing streams were carried out with spatial smoothing (5mm FWHM kernel) and without spatial smoothing. Smoothed and non-smoothed functional data were subjected to ICA-AROMA to regress out motion-related artifacts from the data. Denoised data was then temporally filtered with a highpass filter of 0.01 Hz.

Non-smoothed functional data was registered to an anatomical template using FreeSurfer’s *bbregister* tool, which uses a boundary-based cost function restricted to 6 degrees-of-freedom. Each thalamic nucleus was resampled to the fMRI voxel size. Voxels with < 50% overlap with the original anatomical label were excluded to minimize partial volume effects. Eight nuclei in each hemisphere (Re, Pt, Pc, CL, LD, VAmc, VM, and LSg) that did meet the 50% threshold were excluded from further analysis, leaving 17 nuclei per hemisphere in the final dataset. The timeseries of each thalamic subregion and nucleus was extracted from the resampled labels and demeaned. Thalamic subregions and nuclei timeseries were extracted from non-smoothed functional data to reduce signal overlap between adjacent regions.

Nuisance signals from white matter (WM) and cerebrospinal fluid (CSF) were modelled using aCompCor (Behzadi et al., 2007). WM and CSF partial volume masks were created by resampling WM and CSF FreeSurfer segmentations into functional space using only voxels that overlapped 100% with the original anatomical mask. Thresholded masks that contained less than ten voxels were recreated using a threshold of 95%. WM masks were further eroded by one voxel to reduce partial volume effects. The top five principal components of the WM signal and top five principal components of the CSF signal were used as regressors in subsequent first-level generalized linear models (GLMs).

**Psychotropic Medication Use Analysis**

Current psychotropic medication use was added to our statistical model as a binary variable to assess the potential impact of psychotropic medication use on thalamocortical RSFC (*n* = 209). The following models were used:

Y ~ PTSD Diagnosis + Medication Status + Age + Age^2^ + Sex + (1|Site)

Y ~ PTSD Severity + Medication Status + Age + Age^2^ + Sex + (1|Site)

**Independent Component Analysis Network Construction**

To derive individualized resting-state networks (RSNs), following preprocessing, participant-level ICA was first performed on the denoised, spatially smoothed data using FSL’s *MELODIC ICA* (Beckmann & Smith, 2004). Participant-level ICs were thresholded at *Z* ≥ 2.3 and normalized to MNI space. The spatial overlap between normalized ICs and networks from the Yeo atlas (Yeo et al., 2011) were assessed using Dice coefficients. Since ICA may decompose canonical RSNs into multiple subnetworks, the top six ICs with the highest Dice coefficients for each RSN were identified. Combinations of these six ICs were evaluated using Yeo as a reference atlas, and the combination with the highest Dice coefficient was used to generate a participant-specific network. Only the top six ICs were considered due to a sharp decline in network recapitulation and reduced Dice coefficients in participant networks using six or more ICs (*Figure S1B*).


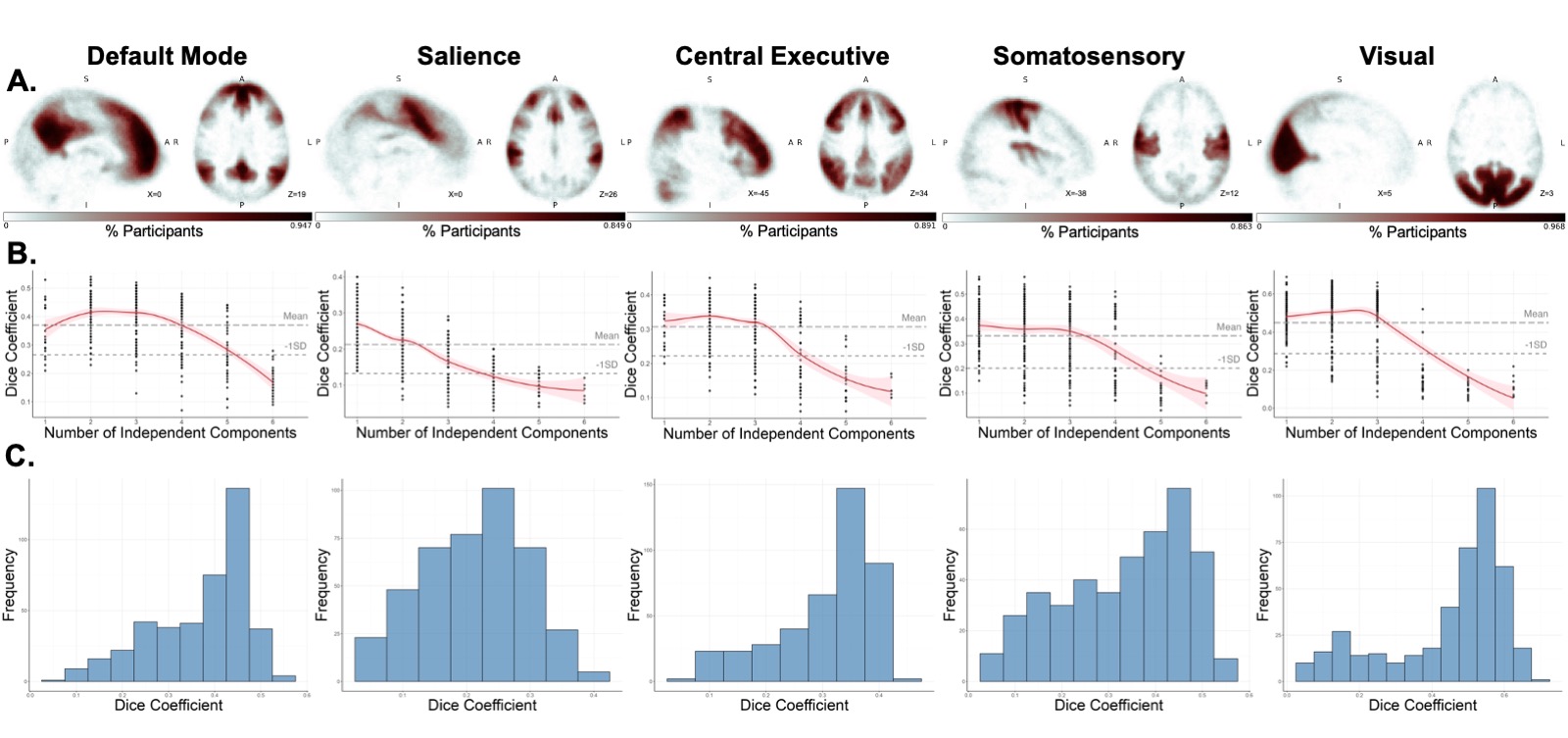
**Figure S1:** ***A)*** *Visualization of the percentage of participants whose ICA network contained each voxel of the brain. Darker regions were included in a higher percentage of participant networks.* ***B)*** *Plots comparing the number of ICs used in each participant's network (x-axis) and the Dice coefficient with the corresponding Yeo network (y-axis). The top dashed line in each graph is the mean Dice coefficient for that network and the bottom dashed line is one standard deviation below the mean.* ***C)*** *Histogram plots for each network are displaying the number of participants binned by Dice coefficients.*

Participants whose individualized RSNs did not faithfully recapitulate Yeo RSNs were excluded from further analysis. Participants with Dice coefficients more than one standard deviation from the mean for a given network were excluded from analysis. The cut-off of one SD was chosen after examining a representative subset of participants' networks and histogram plots of Dice coefficients for each network. Histograms were negatively skewed (*Figure S1C*), and removal of participants below one SD effectively reduced the long negative tail of the distribution. Visual inspection of participants’ network maps confirmed that Dice coefficients below one SD often failed to recapitulate the canonical RSN of interest. The number of participants removed from each network analysis was *n* = 77 for the default mode, *n =* 78 for the salience, *n* = 87 for the somatosensory, *n* = 75 from the central executive, and *n* = 82 for the visual network.

If the ICs used in the network masks represent subnetworks of a common network, they should exhibit strong intercorrelations. Consistent with this expectation, our subnetwork ICs were significantly more correlated than the average IC-IC correlation (r = 0.54, SD = 0.23) for the default mode (r = 0.71, SD = 0.16, t = 8.28), salience (r = 0.70, SD = 0.19, t = 5.78), central executive (r = 0.73, SD = 0.16, t = 9.60), somatosensory (r = 0.77, SD = 0.17, t = 11.13), and visual (r = 0.81, SD = 0.16, t = 14.03) networks.

The average degree of spatial overlap between participant-specific networks and its Yeo network counterpart was consistent with published work on the spatial overlap between RSN atlases (Doucet et al., 2019). Each network displayed an average overlap of 23-49% with the highest average spatial overlap between the Yeo atlas and participants’ visual network. The most common number of ICs (i.e., the statistical mode) used to construct masks for participants’ default mode (mode = 3), somatosensory (mode = 2), and visual (mode = 2) networks aligned with the number of decomposable subnetworks in the Yeo atlas, while participants’ salience (mode = 1) and central executive (mode = 2) networks both averaged one less IC than subnetworks in the Yeo atlas. The number of ICs, their spatial overlap with Yeo networks, and their intercorrelation strength were not associated with PTSD diagnosis or severity (all *p* > 0.05), after adjusting for sex, age, age², and site.

**Seed-Based Functional Connectivity Network Construction**

In addition to constructing individualized RSNs with ICA, individualized RSNs were defined using a seed-based functional connectivity (FC) approach to replicate the ICA network findings. Anatomical and 8mm spherical regions-of-interest (ROIs) were defined as seeds, selected from canonical network areas identified in prior research. The mean time series across these ROIs was extracted and entered into a generalized linear model (GLM) with nuisance signals from white matter (WM) and cerebrospinal fluid (CSF) modelled using aCompCor. The resulting *Z*-statistic maps were thresholded at *Z* ≥ 2.3 and subsequently binarized to define individualized RSNs.

For the default mode network, included spherical ROI seeds were centered in the medial PFC (3, 54, -2), posterior cingulate cortex (0, -52, 26), left inferior parietal cortex (-50, -63, 32), and right inferior parietal cortex (48, -69, 35) (Sharaev et al., 2016). Salience network seeds included spherical ROIs centered in the dorsal anterior cingulate cortex (3, 27, 24), the left anterior insula (-42, 9, 0) and the right anterior insula (42, 9, 0) (Zhao et al., 2022). Somatosensory network seeds included the postcentral gyrus from the probabilistic Harvard-Oxford Cortical Structural Atlas thresholded at 50% and spherical ROIs centered in the left posterior insula (-23, 55, -44) and right posterior insula (23, 55, 44) (Yeo et al., 2011). The central executive and visual networks were not examined with a seed-based FC approach due to a lack of significant findings using ICA-derived networks.

**Appendix V: Volumetric Data**

|  | **Volume (mm^3^)** | | | **Statistic** | | | |
| --- | --- | --- | --- | --- | --- | --- | --- |
| **Region** | **Total** | **PTSD** | **Controls** | **t-statistic** | **Cohen's *d*** | ***p*** | ***p_FDR_*** |
| **Ventral** | | | | | | | |
| Ventral L. | 2997.22 | 2917.06 | 3065.07 | -1.62 | -0.40 | 0.105 | 0.176 |
| Ventral R. | 3110.27 | 3039.19 | 3170.44 | -2.13 | -0.33 | 0.034 | 0.084 |
| VPL L. | 985.62 | 953.53 | 1012.78 | -1.49 | -0.45 | 0.138 | 0.302 |
| VPL R. | 1032.83 | 1008.39 | 1053.52 | -2.35 | -0.29 | 0.019 | 0.120 |
| VLa L. | 612.39 | 597.34 | 625.13 | -1.57 | -0.34 | 0.117 | 0.293 |
| VLa R. | 661.06 | 645.68 | 674.09 | -1.90 | -0.34 | 0.058 | 0.240 |
| VLp L. | 942.66 | 918.52 | 963.10 | -1.39 | -0.38 | 0.166 | 0.345 |
| VLp R. | 957.52 | 935.11 | 976.50 | -1.76 | -0.35 | 0.079 | 0.248 |
| VA L. | 446.89 | 438.27 | 454.18 | -1.70 | -0.23 | 0.091 | 0.260 |
| VA R. | 448.08 | 439.45 | 455.38 | -1.48 | -0.25 | 0.139 | 0.302 |
| VAmc L. | 7.12 | 6.95 | 7.26 | -0.71 | -0.23 | 0.476 | 0.591 |
| VAmc R. | 9.13 | 8.92 | 9.31 | -1.30 | -0.24 | 0.195 | 0.390 |
| VM L. | 2.54 | 2.46 | 2.61 | -0.68 | -0.08 | 0.496 | 0.591 |
| VM R. | 1.65 | 1.66 | 1.64 | -1.15 | 0.01 | 0.249 | 0.416 |
| **Medial** | | | | | | | |
| Medial L. | 1078.98 | 1054.92 | 1099.34 | -1.10 | -0.33 | 0.273 | 0.341 |
| Medial R. | 1115.11 | 1089.22 | 1137.04 | -2.14 | -0.35 | 0.033 | 0.084 |
| MDm L. | 800.72 | 783.20 | 815.55 | -0.87 | -0.32 | 0.387 | 0.578 |
| MDm R. | 826.67 | 806.16 | 844.02 | -2.17 | -0.37 | 0.031 | 0.153 |
| MDl L. | 266.82 | 260.40 | 272.25 | -1.51 | -0.33 | 0.133 | 0.302 |
| MDl R. | 275.71 | 270.13 | 280.43 | -1.84 | -0.27 | 0.067 | 0.248 |
| Re L. | 11.35 | 11.25 | 11.45 | -1.16 | -0.07 | 0.248 | 0.416 |
| Re. R. | 12.59 | 12.78 | 12.44 | -0.80 | 0.09 | 0.426 | 0.591 |
| Pt L. | 0.09 | 0.07 | 0.10 | -0.91 | -0.05 | 0.366 | 0.571 |
| Pt R. | 0.14 | 0.14 | 0.14 | -0.55 | -0.01 | 0.581 | 0.660 |
| **Intralaminar** | | | | | | | |
| Intralaminar L. | 408.20 | 401.75 | 413.66 | 0.12 | -0.21 | 0.907 | 0.907 |
| Intralaminar R. | 421.85 | 417.56 | 425.49 | -0.65 | -0.14 | 0.517 | 0.575 |
| CeM L. | 82.66 | 82.21 | 83.03 | -0.52 | -0.06 | 0.603 | 0.670 |
| CeM R. | 83.96 | 84.14 | 83.80 | -0.86 | 0.02 | 0.393 | 0.578 |
| CM L. | 258.22 | 252.66 | 262.93 | 0.49 | -0.28 | 0.621 | 0.675 |
| CM R. | 265.64 | 261.14 | 269.44 | -0.40 | -0.22 | 0.689 | 0.718 |
| Pf L. | 47.89 | 47.56 | 48.16 | 1.76 | -0.06 | 0.079 | 0.248 |
| Pf R. | 53.18 | 52.34 | 53.88 | -0.07 | -0.16 | 0.940 | 0.940 |
| CL L. | 19.42 | 19.30 | 19.53 | -1.98 | -0.03 | 0.049 | 0.221 |
| CL R. | 18.49 | 19.39 | 17.73 | -0.72 | 0.19 | 0.475 | 0.591 |
| Pc L. | 0.01 | 0.01 | 0.01 | 0.63 | 0.06 | 0.530 | 0.617 |
| Pc R. | 0.60 | 0.55 | 0.63 | -1.20 | -0.22 | 0.233 | 0.416 |
| **Posterior** | | | | | | | |
| Posterior L. | 2245.26 | 2179.71 | 2300.74 | -1.54 | -0.41 | 0.124 | 0.177 |
| Posterior R. | 2380.17 | 2328.36 | 2424.04 | -3.74 | -0.32 | 0.000 | 0.002 |
| PuA L. | 221.65 | 213.81 | 228.29 | -1.82 | -0.44 | 0.070 | 0.248 |
| PuA R. | 231.22 | 225.92 | 235.70 | -3.26 | -0.31 | 0.001 | 0.015 |
| PuM L. | 1203.43 | 1165.10 | 1235.88 | -1.62 | -0.42 | 0.106 | 0.279 |
| PuM R. | 1298.43 | 1269.57 | 1322.86 | -3.61 | -0.31 | 0.000 | 0.009 |
| PuL L. | 189.60 | 185.27 | 193.26 | -0.68 | -0.20 | 0.495 | 0.591 |
| PuL R. | 194.16 | 193.18 | 194.99 | -1.17 | -0.05 | 0.242 | 0.416 |
| PuI L. | 231.57 | 222.54 | 239.22 | -1.16 | -0.39 | 0.249 | 0.416 |
| PuI R. | 245.72 | 238.05 | 252.20 | -3.83 | -0.32 | 0.000 | 0.008 |
| LGN L. | 287.28 | 279.47 | 293.89 | -0.74 | -0.34 | 0.461 | 0.591 |
| LGN R. | 292.41 | 281.92 | 301.29 | -3.44 | -0.44 | 0.001 | 0.011 |
| MGN L. | 92.53 | 94.16 | 91.15 | -0.75 | 0.14 | 0.452 | 0.591 |
| MGN R. | 104.13 | 105.47 | 103.00 | -0.47 | 0.10 | 0.640 | 0.680 |
| LSg L. | 19.19 | 19.37 | 19.05 | -0.32 | 0.04 | 0.748 | 0.764 |
| LSg R. | 14.11 | 14.25 | 13.99 | -0.83 | 0.04 | 0.405 | 0.579 |
| **Lateral** | | | | | | | |
| Lateral L. | 177.21 | 172.17 | 181.48 | -2.46 | -0.28 | 0.014 | 0.071 |
| Lateral R. | 176.05 | 173.45 | 178.26 | -1.95 | -0.14 | 0.052 | 0.103 |
| LP L. | 145.76 | 141.47 | 149.40 | -2.54 | -0.33 | 0.011 | 0.089 |
| LP R. | 143.83 | 140.52 | 146.63 | -2.22 | -0.24 | 0.027 | 0.151 |
| LD L. | 31.45 | 30.71 | 32.08 | -1.68 | -0.12 | 0.093 | 0.260 |
| LD R. | 32.23 | 32.93 | 31.63 | -0.92 | 0.11 | 0.358 | 0.571 |
| **Anterior** | | | | | | | |
| AV L. | 154.70 | 152.14 | 156.88 | -2.51 | -0.17 | 0.012 | 0.089 |
| AV R. | 169.85 | 167.77 | 171.61 | -2.66 | -0.13 | 0.008 | 0.082 |

**Table S5:** Volumetric data for each thalamic subregion and nucleus. Statistical significance of volumetric differences between diagnostic groups was tested while controlling for sex, age, age^2^  (fixed effects), and site (random effect) and were corrected for multiple comparisons using the false discovery rate (FDR) at *q*=0.05. Corrections were applied separately for thalamic subregions (10 comparisons) and thalamic nuclei (50 comparisons). The anterior subregion is composed of only one nucleus, the anteroventral (AV) nucleus, and was included in the nuclei analysis and not in the subregion analysis. Statistically significant results are highlighted. Negative t-statistics and Cohen’s *d* values represent lower volumes in PTSD compared to controls.

**Appendix VI: Sex-Stratified Analyses**

| **ROI** | **Size (voxels)** | **Peak z-score** | **Peak x (mm)** | **Peak y (mm)** | **Peak z (mm)** | **Harvard Oxford Cortical Atlas Label** |
| --- | --- | --- | --- | --- | --- | --- |
| **PTSD < Controls** | | | | | | |
| **Posterior L.** | 710 | -5.37 | 12 | -6 | 32 | Cingulate Gyrus, anterior division |
|  | 33 | -4.80 | 8 | 20 | 28 | Cingulate Gyrus, anterior division |
|  | 13 | -3.38 | -2 | 32 | 42 | Paracingulate Gyrus |
| LGN L. | 12 | -5.45 | -42 | 4 | 28 | Precentral Gyrus |
| MGN L. | 34 | -4.51 | -48 | 34 | 16 | Inferior Frontal Gyrus, pars triangularis |
| PuI L. | 16 | -5.42 | 48 | -2 | 40 | Precentral Gyrus |
|  | 14 | -5.10 | 8 | -2 | 36 | Cingulate Gyrus, anterior division |
| **Posterior R.** | 11 | -3.72 | 10 | 12 | 52 | Paracingulate Gyrus |
| PuI R. | 1144 | -5.63 | 4 | 4 | 30 | Cingulate Gyrus, anterior division |
|  | 501 | -5.60 | 10 | -42 | 50 | Precuneus Cortex |
|  | 213 | -5.59 | 32 | 0 | 62 | Middle Frontal Gyrus |
|  | 257 | -5.45 | -10 | -30 | 68 | Precentral Gyrus |
|  | 36 | -5.39 | 44 | 12 | 20 | Inferior Frontal Gyrus, pars opercularis |
|  | 79 | -5.30 | 62 | -36 | 26 | Supramarginal Gyrus, posterior division |
|  | 67 | -5.28 | 40 | -44 | 34 | Supramarginal Gyrus, posterior division |
|  | 60 | -5.24 | -48 | 0 | 38 | Precentral Gyrus |
|  | 94 | -5.23 | 42 | -48 | 52 | Superior Parietal Lobule |
|  | 12 | -5.22 | 26 | 6 | 42 | Superior Frontal Gyrus |
|  | 37 | -5.14 | -42 | -46 | 52 | Superior Parietal Lobule |
|  | 34 | -5.13 | -2 | -52 | 66 | Precuneus Cortex |
|  | 52 | -5.06 | 26 | -54 | 40 | Superior Parietal Lobule |
|  | 15 | -5.05 | 62 | -24 | 44 | Supramarginal Gyrus, anterior division |
|  | 17 | -5.05 | -34 | -54 | 58 | Superior Parietal Lobule |
|  | 11 | -5.04 | 50 | -42 | -2 | Middle Temporal Gyrus, temporooccipital part |
|  | 14 | -5.02 | -58 | -30 | 38 | Supramarginal Gyrus, anterior division |
|  | 147 | -4.99 | 10 | 22 | 26 | Cingulate Gyrus, anterior division |
|  | 12 | -4.99 | 50 | -36 | 10 | Supramarginal Gyrus, posterior division |
| **Ventral L.** | - | - | - | - | - | - |
| VA L. | 16 | -5.33 | -26 | -58 | 34 | Angular Gyrus |
| **PTSD > Controls** | | | | | | |
| **Posterior R.** | - | - | - | - | - | - |
| MGN R. | 16 | 4.79 | 26 | -30 | 50 | Precentral Gyrus |
|  | 22 | 4.71 | 58 | -8 | 36 | Postcentral Gyrus |
|  | 38 | 3.96 | -50 | -8 | 38 | Precentral Gyrus |

**Table S6:** Associations between thalamic RSFC and PTSD diagnosis in males only.

| **ROI** | **Size (voxels)** | **Peak z-score** | **Peak x (mm)** | **Peak y (mm)** | **Peak z (mm)** | **Harvard Oxford Cortical Atlas Label** |
| --- | --- | --- | --- | --- | --- | --- |
| **Medial L.** | 12 | -5.20 | 30 | -88 | 18 | Lateral Occipital Cortex, superior division |
| MDm L. | 150 | -5.83 | 30 | -90 | 18 | Occipital Pole |
|  | 12 | -5.17 | -8 | -90 | 14 | Occipital Pole |
| **Medial R.** | 16 | -4.80 | 46 | -76 | -2 | Lateral Occipital Cortex, inferior division |
|  | 32 | -4.79 | 6 | 6 | 60 | Juxtapositional Lobule Cortex (formerly Supplementary Motor Cortex) |
| MDl R. | 139 | -5.94 | 54 | -2 | 48 | Precentral Gyrus |
|  | 30 | -5.74 | 44 | -24 | 32 | Supramarginal Gyrus, anterior division |
|  | 32 | -5.47 | 46 | -38 | 62 | Postcentral Gyrus |
|  | 16 | -5.33 | 48 | -70 | -2 | Lateral Occipital Cortex, inferior division |
|  | 22 | -5.21 | 30 | -80 | -12 | Occipital Fusiform Gyrus |
|  | 19 | -5.19 | 40 | -4 | 58 | Precentral Gyrus |
|  | 20 | -5.12 | 44 | -32 | 46 | Postcentral Gyrus |
| MDm R. | 29 | -5.00 | 44 | -70 | 4 | Lateral Occipital Cortex, inferior division |
|  | 199 | -4.99 | 0 | -14 | 72 | Precentral Gyrus |
|  | 28 | -4.87 | 6 | 4 | 62 | Juxtapositional Lobule Cortex (formerly Supplementary Motor Cortex) |
|  | 23 | -4.70 | 42 | -74 | 22 | Lateral Occipital Cortex, superior division |
|  | 41 | -4.53 | 8 | -20 | 66 | Precentral Gyrus |
|  | 28 | -4.37 | 4 | -48 | 56 | Precuneous Cortex |
| **Posterior L.** | 261 | -5.66 | 6 | 6 | 46 | Juxtapositional Lobule Cortex (formerly Supplementary Motor Cortex) |
|  | 147 | -5.56 | 4 | -6 | 70 | Juxtapositional Lobule Cortex (formerly Supplementary Motor Cortex) |
| LGN L. | 35 | -5.45 | -42 | 4 | 26 | Precentral Gyrus |
|  | 17 | -5.32 | 6 | 8 | 32 | Cingulate Gyrus, anterior division |
|  | 20 | -5.16 | 8 | 14 | 48 | Paracingulate Gyrus |
|  | 21 | -5.05 | -56 | 14 | 2 | Inferior Frontal Gyrus, pars opercularis |
|  | 14 | -4.88 | 4 | 6 | 66 | Juxtapositional Lobule Cortex (formerly Supplementary Motor Cortex) |
| PuM L. | 140 | -5.61 | 4 | -20 | 72 | Precentral Gyrus |
|  | 28 | -5.44 | 4 | -26 | 62 | Precentral Gyrus |
|  | 33 | -5.07 | 22 | -22 | 70 | Precentral Gyrus |
| **Posterior R.** | 23 | -5.34 | 4 | -12 | 68 | Juxtapositional Lobule Cortex (formerly Supplementary Motor Cortex) |
| PuA R. | 18 | -4.73 | 36 | -90 | 16 | Occipital Pole |
| PuI R. | 1012 | -5.91 | -26 | -6 | 44 | Precentral Gyrus |
|  | 572 | -5.81 | 26 | 6 | 42 | Superior Frontal Gyrus |
|  | 1571 | -5.41 | -28 | -54 | 70 | Superior Parietal Lobule |
|  | 1395 | -5.37 | 2 | -10 | 52 | Juxtapositional Lobule Cortex (formerly Supplementary Motor Cortex) |
|  | 295 | -5.29 | -12 | -40 | 76 | Postcentral Gyrus |
|  | 272 | -5.14 | 4 | -28 | 48 | Precentral Gyrus |
|  | 64 | -5.05 | 44 | -12 | 40 | Precentral Gyrus |
|  | 65 | -5.03 | 46 | 4 | 38 | Precentral Gyrus |
|  | 17 | -5.02 | -12 | -24 | 44 | Precentral Gyrus |
|  | 55 | -4.97 | -32 | 8 | 2 | Insular Cortex |
|  | 12 | -4.92 | -48 | -2 | 4 | Central Opercular Cortex |
|  | 218 | -4.84 | 34 | 2 | -4 | Insular Cortex |
|  | 362 | -4.82 | 18 | -54 | 56 | Superior Parietal Lobule |
|  | 36 | -4.79 | 0 | -68 | -8 | Lingual Gyrus |
|  | 50 | -4.65 | 52 | 6 | 12 | Precentral Gyrus |
|  | 95 | -4.62 | 60 | -20 | 22 | Supramarginal Gyrus, anterior division |
|  | 13 | -4.60 | 44 | -2 | 28 | Precentral Gyrus |
|  | 157 | -4.54 | 64 | -32 | 26 | Parietal Opercular Cortex |
|  | 340 | -4.40 | 30 | -44 | 46 | Superior Parietal Lobule |
|  | 63 | -4.34 | 2 | -32 | 70 | Precentral Gyrus |
|  | 11 | -4.29 | -32 | -34 | 62 | Postcentral Gyrus |
|  | 10 | -4.27 | -50 | 10 | 38 | Middle Frontal Gyrus |
|  | 17 | -4.13 | 42 | -50 | 54 | Superior Parietal Lobule |
|  | 121 | -4.13 | 64 | -24 | 44 | Supramarginal Gyrus, anterior division |
|  | 11 | -3.91 | 56 | -4 | 32 | Precentral Gyrus |
|  | 97 | -3.88 | 32 | -56 | 48 | Superior Parietal Lobule |
|  | 12 | -3.76 | -40 | -26 | 64 | Postcentral Gyrus |
| PuM R. | 90 | -5.57 | 26 | -90 | 30 | Occipital Pole |
|  | 55 | -5.29 | 4 | -10 | 68 | Juxtapositional Lobule Cortex (formerly Supplementary Motor Cortex) |
|  | 33 | -5.11 | 26 | -84 | 20 | Lateral Occipital Cortex, superior division |

**Table S7:** Associations between thalamic RSFC and PTSD severity in males only.

| **ROI** | **Size (voxels)** | **Peak z-score** | **Peak x (mm)** | **Peak y (mm)** | **Peak z (mm)** | **Harvard Oxford Cortical Atlas Label** |
| --- | --- | --- | --- | --- | --- | --- |
| **PTSD < Controls** | | | | | | |
| **Posterior L.** | 418 | -5.35 | 6 | 28 | 44 | Paracingulate Gyrus |
|  | 58 | -4.07 | 0 | 0 | 70 | Juxtapositional Lobule Cortex (formerly Supplementary Motor Cortex) |
| MGN L. | 10 | -3.43 | -46 | 34 | 16 | Inferior Frontal Gyrus, pars triangularis |
| **Posterior R.** | 11 | -5.01 | 8 | 12 | 52 | Paracingulate Gyrus |
| PuI R. | 181 | -4.04 | 12 | 16 | 54 | Superior Frontal Gyrus |
|  | 22 | -3.96 | 4 | 4 | 64 | Juxtapositional Lobule Cortex (formerly Supplementary Motor Cortex) |
|  | 20 | -3.48 | 4 | 28 | 36 | Paracingulate Gyrus |

**Table S8:** Associations between thalamic RSFC and PTSD diagnosis in females only.

| **ROI** | **Size (voxels)** | **Peak z-score** | **Peak x (mm)** | **Peak y (mm)** | **Peak z (mm)** | **Harvard Oxford Cortical Atlas Label** |
| --- | --- | --- | --- | --- | --- | --- |
| **Posterior R.** | - | - | - | - | - | - |
| PuI R. | 300 | -4.97 | -34 | -54 | 56 | Superior Parietal Lobule |
|  | 169 | -4.88 | -4 | -36 | 70 | Postcentral Gyrus |
|  | 960 | -4.78 | 24 | -56 | 40 | Lateral Occipital Cortex, superior division |
|  | 60 | -4.78 | 30 | -48 | 64 | Superior Parietal Lobule |
|  | 70 | -4.76 | 32 | 0 | 60 | Middle Frontal Gyrus |
|  | 56 | -4.65 | 20 | 8 | 40 | Superior Frontal Gyrus |
|  | 19 | -4.53 | 30 | 8 | 58 | Middle Frontal Gyrus |
|  | 337 | -4.37 | 12 | 16 | 54 | Superior Frontal Gyrus |
|  | 143 | -4.35 | -10 | 18 | 26 | Cingulate Gyrus, anterior division |
|  | 359 | -4.34 | -46 | -10 | 40 | Precentral Gyrus |
|  | 353 | -4.29 | -32 | 2 | 58 | Middle Frontal Gyrus |
|  | 110 | -4.27 | 4 | -4 | 44 | Cingulate Gyrus, anterior division |
|  | 157 | -4.16 | -50 | -38 | 48 | Supramarginal Gyrus, anterior division |
|  | 24 | -3.89 | -16 | -10 | 34 | Cingulate Gyrus, anterior division |
|  | 11 | -3.81 | 38 | 0 | 28 | Precentral Gyrus |
|  | 71 | -3.75 | 62 | -34 | 30 | Parietal Opercular Cortex |
|  | 35 | -3.75 | -4 | -12 | 36 | Cingulate Gyrus, anterior division |
|  | 60 | -3.74 | -58 | -28 | 36 | Supramarginal Gyrus, anterior division |
|  | 20 | -3.73 | 22 | -8 | 50 | Superior Frontal Gyrus |
|  | 18 | -3.71 | 70 | -20 | 28 | Supramarginal Gyrus, anterior division |
|  | 32 | -3.65 | 0 | -66 | -8 | Lingual Gyrus |
|  | 12 | -3.59 | 6 | -42 | 60 | Postcentral Gyrus |
|  | 12 | -3.37 | -14 | -38 | 56 | Postcentral Gyrus |
|  | 19 | -3.29 | -2 | 10 | 50 | Paracingulate Gyrus |

**Table S9:** Associations between thalamic RSFC and PTSD severity in females only.

**Appendix VIII: Psychotropic Medication Status Analysis**

| **ROI** | **Size (voxels)** | **Peak z-score** | **Peak x (mm)** | **Peak y (mm)** | **Peak z (mm)** | **Harvard Oxford Cortical Atlas Label** |
| --- | --- | --- | --- | --- | --- | --- |
| **PTSD < Controls** | | | | | | |
| **Posterior L.** | 465 | 5.75 | 4 | 12 | 54 | Superior Frontal Gyrus |
|  | 13 | 4.19 | -6 | 8 | 38 | Cingulate Gyrus, anterior division |
|  | 80 | 3.84 | 8 | 8 | 28 | Cingulate Gyrus, anterior division |
|  | 18 | 3.47 | 6 | 28 | 42 | Paracingulate Gyrus |
| MGN L. | 34 | 4.56 | -50 | 30 | 20 | Inferior Frontal Gyrus, pars triangularis |
| **Posterior R.** | 11 | 4.86 | 8 | 10 | 54 | Paracingulate Gyrus |
| PuI R. | 149 | 4.86 | -2 | 32 | 32 | Paracingulate Gyrus |
|  | 16 | 4.71 | -34 | -54 | 56 | Superior Parietal Lobule |
|  | 12 | 4.47 | 46 | -42 | -4 | Middle Temporal Gyrus, posterior division |
|  | 33 | 4.45 | -40 | -56 | 52 | Angular Gyrus |
|  | 95 | 4.43 | -16 | 0 | 52 | Superior Frontal Gyrus |
|  | 322 | 4.43 | 12 | 4 | 60 | Juxtapositional Lobule Cortex (formerly Supplementary Motor Cortex) |
|  | 70 | 4.42 | -10 | 6 | 28 | Cingulate Gyrus, anterior division |
|  | 56 | 4.09 | 60 | -36 | 28 | Supramarginal Gyrus, posterior division |
|  | 20 | 4.06 | 18 | -32 | 44 | Precentral Gyrus |
|  | 195 | 4.03 | 16 | -52 | 56 | Superior Parietal Lobule |
|  | 40 | 4.00 | 28 | -44 | 42 | Superior Parietal Lobule |
|  | 18 | 3.94 | 6 | 0 | 46 | Cingulate Gyrus, anterior division |
|  | 18 | 3.87 | 32 | 0 | 62 | Middle Frontal Gyrus |
|  | 60 | 3.77 | 42 | -50 | 54 | Superior Parietal Lobule |
|  | 42 | 3.74 | 2 | 18 | 60 | Superior Frontal Gyrus |
|  | 12 | 3.63 | 46 | -52 | 42 | Angular Gyrus |
|  | 32 | 3.63 | 40 | -44 | 34 | Supramarginal Gyrus, posterior division |
|  | 11 | 3.61 | -54 | -32 | 36 | Supramarginal Gyrus, anterior division |
|  | 25 | 3.55 | -48 | -6 | 42 | Precentral Gyrus |
|  | 10 | 3.51 | 58 | -28 | 46 | Supramarginal Gyrus, anterior division |
|  | 11 | 3.46 | 46 | 12 | 26 | Inferior Frontal Gyrus, pars opercularis |
|  | 10 | 3.34 | 8 | -42 | 68 | Postcentral Gyrus |
|  | 11 | 3.20 | -2 | -56 | 62 | Precuneous Cortex |
| **Ventral L.** | - | - | - | - | - | - |
| VA L. | 11 | 3.59 | -28 | -56 | 36 | Superior Parietal Lobule |
| **PTSD > Controls** | | | | | | |
| **Posterior R.** | - | - | - | - | - | - |
| MGN R. | 35 | 4.83 | -52 | -8 | 42 | Precentral Gyrus |
|  | 11 | 4.10 | 28 | -30 | 50 | Postcentral Gyrus |
|  | 14 | 3.94 | 64 | -8 | 40 | Postcentral Gyrus |

**Table S10:** Associations between thalamic RSFC and PTSD diagnosis when adjusting for psychotropic medication use.

| **ROI** | **Size (voxels)** | **Peak z-score** | **Peak x (mm)** | **Peak y (mm)** | **Peak z (mm)** | **Harvard Oxford Cortical Atlas Label** |
| --- | --- | --- | --- | --- | --- | --- |
| **Posterior L.** | 66 | -3.93 | 4 | 2 | 58 | Juxtapositional Lobule Cortex (formerly Supplementary Motor Cortex) |
|  | 35 | -3.43 | 10 | 8 | 28 | Cingulate Gyrus, anterior division |
| LGN L. | 17 | -4.68 | 8 | 8 | 34 | Cingulate Gyrus, anterior division |
|  | 12 | -4.48 | 2 | 6 | 62 | Juxtapositional Lobule Cortex (formerly Supplementary Motor Cortex) |
|  | 14 | -3.64 | 8 | 14 | 48 | Paracingulate Gyrus |
| PuM L. | 25 | -4.34 | 18 | -22 | 68 | Precentral Gyrus |
| **Posterior R.** | - | - | - | - | - | - |
| PuI R. | 461 | -4.32 | -34 | -54 | 56 | Superior Parietal Lobule |
|  | 116 | -4.14 | 12 | 14 | 54 | Superior Frontal Gyrus |
|  | 326 | -4.06 | 28 | -44 | 38 | Superior Parietal Lobule |
|  | 24 | -3.96 | -16 | 2 | 52 | Superior Frontal Gyrus |
|  | 17 | -3.83 | 0 | -64 | -8 | Cerebellar Lobule V |
|  | 82 | -3.81 | -10 | 18 | 28 | Cingulate Gyrus, anterior division |
|  | 32 | -3.76 | -42 | -16 | 28 | Postcentral Gyrus |
|  | 14 | -3.72 | -28 | -8 | 46 | Precentral Gyrus |
|  | 191 | -3.69 | 10 | -62 | 62 | Lateral Occipital Cortex, superior division |
|  | 12 | -3.64 | -32 | 8 | 2 | Insular Cortex |
|  | 139 | -3.60 | 40 | -44 | 34 | Supramarginal Gyrus, posterior division |
|  | 34 | -3.58 | 8 | 26 | 34 | Paracingulate Gyrus |
|  | 20 | -3.52 | -2 | -42 | 68 | Postcentral Gyrus |
|  | 30 | -3.48 | -52 | -2 | 40 | Precentral Gyrus |
|  | 10 | -3.43 | 20 | 8 | 40 | Paracingulate gyrus |
|  | 29 | -3.42 | -14 | -56 | 74 | Superior Parietal Lobule |
|  | 13 | -3.41 | 34 | 2 | 62 | Middle Frontal Gyrus |
|  | 24 | -3.41 | -2 | 32 | 32 | Paracingulate Gyrus |
|  | 13 | -3.27 | 46 | -50 | 56 | Angular Gyrus |
| PuM R. | 25 | -4.08 | 24 | -90 | 32 | Occipital Pole |
| **Medial R.** | 22 | -3.79 | 4 | 4 | 60 | Juxtapositional Lobule Cortex (formerly Supplementary Motor Cortex) |
| MDl R. | 32 | -3.86 | 52 | -4 | 48 | Precentral Gyrus |
| MDm R. | 113 | -4.65 | -12 | -24 | 60 | Precentral Gyrus |
|  | 39 | -4.13 | 8 | -16 | 64 | Precentral Gyrus |
|  | 12 | -3.20 | -4 | -8 | 76 | Juxtapositional Lobule Cortex (formerly Supplementary Motor Cortex) |

**Table S11:** Associations between thalamic RSFC and PTSD severity when adjusting for psychotropic medication use.

**Appendix VIII: Comparison Using Only Trauma-Exposed Controls**

| **ROI** | **Size (voxels)** | **Peak z-score** | **Peak x (mm)** | **Peak y (mm)** | **Peak z (mm)** | **Harvard Oxford Cortical Atlas Label** |
| --- | --- | --- | --- | --- | --- | --- |
| **PTSD < TEC** | | | | | | |
| **Posterior L.** | - | - | - | - | - | - |
| MGN L. | 34 | -5.70 | -48 | 34 | 16 | Inferior Frontal Gyrus, pars triangularis |
| PuI L. | 14 | -5.65 | 10 | -2 | 36 | Cingulate Gyrus, anterior division |
|  | 16 | -5.42 | 48 | -2 | 40 | Precentral Gyrus |
| **Posterior R.** | - | - | - | - | - | - |
| PuI R. | 1243 | -5.75 | 6 | 4 | 62 | Juxtapositional Lobule Cortex (formerly Supplementary Motor Cortex) |
|  | 518 | -5.62 | 4 | -44 | 56 | Precuneus Cortex |
|  | 36 | -5.61 | 44 | 12 | 20 | Inferior Frontal Gyrus, pars opercularis |
|  | 153 | -5.60 | 12 | 22 | 26 | Cingulate Gyrus, anterior division |
|  | 213 | -5.59 | 32 | 0 | 62 | Middle Frontal Gyrus |
|  | 78 | -5.55 | 62 | -36 | 28 | Supramarginal Gyrus, posterior division |
|  | 257 | -5.53 | -8 | -32 | 68 | Precentral Gyrus |
|  | 44 | -5.40 | 24 | -56 | 40 | Lateral Occipital Cortex, superior division |
|  | 67 | -5.37 | 40 | -44 | 34 | Supramarginal Gyrus, posterior division |
|  | 99 | -5.32 | 46 | -44 | 54 | Supramarginal Gyrus, posterior division |
|  | 62 | -5.32 | -48 | 0 | 38 | Precentral Gyrus |
|  | 17 | -5.24 | -34 | -54 | 58 | Superior Parietal Lobule |
|  | 12 | -5.22 | 26 | 6 | 42 | Superior Frontal Gyrus |
|  | 34 | -5.15 | -2 | -54 | 64 | Precuneus Cortex |
|  | 38 | -5.14 | -42 | -46 | 52 | Superior Parietal Lobule |
|  | 12 | -5.13 | 48 | -42 | -2 | Middle Temporal Gyrus, temporooccipital part |
|  | 15 | -5.07 | 62 | -24 | 44 | Supramarginal Gyrus, anterior division |
|  | 14 | -5.06 | -54 | -32 | 34 | Supramarginal Gyrus, anterior division |
|  | 12 | -4.99 | 50 | -36 | 10 | Supramarginal Gyrus, posterior division |
| **Ventral L.** | - | - | - | - | - | - |
| VA L. | 16 | -5.56 | -26 | -56 | 36 | Superior Parietal Lobule |
| **PTSD > TEC** | | | | | | |
| **Posterior R.** | - | - | - | - | - | - |
| MGN R. | 20 | 4.23 | 60 | -8 | 36 | Postcentral Gyrus |
|  | 16 | 4.18 | 28 | -30 | 50 | Postcentral Gyrus |

**Table S12:** Associations between thalamic RSFC and PTSD diagnosis when excluding trauma-naive participants.

| **ROI** | **Size (voxels)** | **Peak z-score** | **Peak x (mm)** | **Peak y (mm)** | **Peak z (mm)** | **Harvard Oxford Cortical Atlas Label** |
| --- | --- | --- | --- | --- | --- | --- |
| **Medial L.** | - | - | - | - | - | - |
| MDm L. | 150 | -5.83 | 30 | -90 | 18 | Occipital Pole |
|  | 12 | -5.17 | -8 | -90 | 14 | Occipital Pole |
| **Medial R.** | - | - | - | - | - | - |
| MDl R. | 139 | -5.97 | 54 | -2 | 48 | Precentral Gyrus |
|  | 30 | -5.74 | 44 | -24 | 32 | Supramarginal Gyrus, anterior division |
|  | 32 | -5.47 | 46 | -38 | 62 | Postcentral Gyrus |
|  | 16 | -5.33 | 48 | -70 | -2 | Lateral Occipital Cortex, inferior division |
|  | 22 | -5.21 | 30 | -80 | -12 | Occipital Fusiform Gyrus |
|  | 19 | -5.19 | 40 | -4 | 58 | Precentral Gyrus |
|  | 20 | -5.12 | 44 | -32 | 46 | Postcentral Gyrus |
| MDm R. | 23 | -5.62 | 42 | -72 | 22 | Lateral Occipital Cortex, superior division |
|  | 199 | -5.52 | -12 | -22 | 62 | Precentral Gyrus |
|  | 41 | -5.39 | 10 | -20 | 66 | Precentral Gyrus |
|  | 28 | -5.34 | 6 | 4 | 60 | Juxtapositional Lobule Cortex (formerly Supplementary Motor Cortex) |
|  | 29 | -5.24 | 0 | -46 | 58 | Precuneous Cortex |
|  | 29 | -5.23 | 46 | -74 | -2 | Lateral Occipital Cortex, inferior division |
| **Posterior L.** | - | - | - | - | - | - |
| PuM L. | 140 | -5.61 | 4 | -20 | 72 | Precentral Gyrus |
|  | 28 | -5.50 | 4 | -26 | 62 | Precentral Gyrus |
|  | 33 | -5.33 | 20 | -22 | 68 | Precentral Gyrus |
| **Posterior R.** | - | - | - | - | - | - |
| PuA R. | 18 | -5.78 | 36 | -90 | 16 | Occipital Pole |
| PuI R. | 2831 | -6.37 | -28 | -8 | 46 | Precentral Gyrus |
|  | 590 | -6.12 | 26 | 6 | 42 | Superior Frontal Gyrus |
|  | 1652 | -5.87 | 10 | 10 | 52 | Paracingulate Gyrus |
|  | 1334 | -5.63 | 30 | -48 | 64 | Superior Parietal Lobule |
|  | 341 | -5.41 | -18 | -44 | 74 | Postcentral Gyrus |
|  | 273 | -5.34 | 34 | -32 | 44 | Postcentral Gyrus |
|  | 55 | -5.30 | -32 | 10 | 2 | Insular Cortex |
|  | 111 | -5.29 | 62 | -18 | 22 | Postcentral Gyrus |
|  | 136 | -5.25 | 64 | -32 | 26 | Parietal Opercular Cortex |
|  | 51 | -5.18 | 56 | 12 | 14 | Inferior Frontal Gyrus, pars opercularis |
|  | 45 | -5.08 | -28 | -4 | 66 | Superior Frontal Gyrus |
|  | 12 | -5.08 | -18 | -50 | 50 | Superior Parietal Lobule |
|  | 68 | -5.06 | 44 | -14 | 40 | Precentral Gyrus |
|  | 68 | -5.06 | 44 | 4 | 36 | Precentral Gyrus |
|  | 25 | -5.05 | 44 | 0 | 30 | Precentral Gyrus |
|  | 31 | -5.04 | -40 | -26 | 64 | Postcentral Gyrus |
|  | 36 | -4.78 | 0 | -68 | -6 | Lingual Gyrus |
|  | 12 | -4.77 | -48 | -2 | 4 | Central Opercular Cortex |
|  | 10 | -4.60 | -30 | -32 | 62 | Postcentral Gyrus |
|  | 215 | -4.52 | 34 | 2 | -4 | Insular Cortex |
|  | 23 | -4.34 | 20 | 20 | 50 | Superior Frontal Gyrus |
| PuM R. | 90 | -6.15 | 24 | -90 | 32 | Occipital Pole |
|  | 55 | -5.71 | 4 | -12 | 68 | Juxtapositional Lobule Cortex (formerly Supplementary Motor Cortex) |
|  | 33 | -5.17 | 32 | -86 | 18 | Lateral Occipital Cortex, superior division |
| **Ventral L.** | - | - | - | - | - | - |
| VPL L. | 16 | -5.07 | 8 | 12 | 30 | Cingulate Gyrus, anterior division |

**Table S13:** Associations between thalamic RSFC and PTSD severity when excluding trauma-naive participants.

**Appendix IX: Supplementary Figures**

**Figure S2:** Whole-brain RSFC maps showing significant associations between PTSD diagnosis and thalamocortical connectivity. Bar graphs display the average Fisher *z*-transformed thalamocortical functional connectivity extracted from the peak voxel for the PTSD (red) and control (grey) groups. **(A)** The right MGN exhibited stronger RSFC with the sensorimotor cortex, while the **(B)** right PuI and **(C)** left VA nuclei exhibited weaker RSFC in PTSD compared to controls.

******Figure S3:** Whole-brain RSFC maps showing significant associations between PTSD symptom severity and thalamocortical connectivity. Scatter plots display the correlation between PTSD severity and Fisher *z*-transformed thalamocortical functional connectivity extracted from the peak voxel (higher group-level absolute *z*-score). **(A)** Right posterior thalamic subregion and **(B)** right medial thalamic subregion exhibit weaker RSFC with increasing symptom severity.

**Appendix X: Supplementary References**

Beckmann, C. F., & Smith, S. M. (2004). Probabilistic independent component analysis for functional magnetic resonance imaging. IEEE Transactions on Medical Imaging, 23(2), 137–152. https://doi.org/10.1109/TMI.2003.822821

Behzadi, Y., Restom, K., Liau, J., & Liu, T. T. (2007). A Component Based Noise Correction Method (CompCor) for BOLD and Perfusion Based fMRI. NeuroImage, 37(1), 90–101. https://doi.org/10.1016/j.neuroimage.2007.04.042

Doucet, G. E., Lee, W. H., & Frangou, S. (2019). Evaluation of the spatial variability in the major resting‐state networks across human brain functional atlases. Human Brain Mapping, 40(15), 4577–4587. https://doi.org/10.1002/hbm.24722

Jenkinson, M., Beckmann, C. F., Behrens, T. E. J., Woolrich, M. W., & Smith, S. M. (2012). FSL. NeuroImage, 62(2), 782–790. https://doi.org/10.1016/j.neuroimage.2011.09.015

Sharaev, M. G., Zavyalova, V. V., Ushakov, V. L., Kartashov, S. I., & Velichkovsky, B. M. (2016). Effective Connectivity within the Default Mode Network: Dynamic Causal Modeling of Resting-State fMRI Data. Frontiers in Human Neuroscience, 10. https://doi.org/10.3389/fnhum.2016.00014

Yeo, B. T., Krienen, F. M., Sepulcre, J., Sabuncu, M. R., Lashkari, D., Hollinshead, M., Roffman, J. L., Smoller, J. W., Zöllei, L., Polimeni, J. R., Fischl, B., Liu, H., & Buckner, R. L. (2011). The organization of the human cerebral cortex estimated by intrinsic functional connectivity. Journal of Neurophysiology, 106(3), 1125–1165. https://doi.org/10.1152/jn.00338.2011

Zhao, L., Bo, Q., Zhang, Z., Chen, Z., Wang, Y., Zhang, D., Li, T., Yang, N., Zhou, Y., & Wang, C. (2022). Altered Dynamic Functional Connectivity in Early Psychosis Between the Salience Network and Visual Network. Neuroscience, 491, 166–175. https://doi.org/10.1016/j.neurosciene
